# Phenylpropanoids Accumulation in Eggplant Fruit: Characterization of Biosynthetic Genes and Regulation by a MYB Transcription Factor

**DOI:** 10.3389/fpls.2015.01233

**Published:** 2016-01-28

**Authors:** Teresa Docimo, Gianluca Francese, Alessandra Ruggiero, Giorgia Batelli, Monica De Palma, Laura Bassolino, Laura Toppino, Giuseppe L. Rotino, Giuseppe Mennella, Marina Tucci

**Affiliations:** ^1^Consiglio Nazionale delle Ricerche, Istituto di Bioscienze e BiorisorseUOS Portici, Italy; ^2^Consiglio per la Ricerca in Agricoltura e l’Analisi dell’Economia Agraria, Centro di Ricerca per l’OrticolturaPontecagnano, Italy; ^3^Consiglio per la Ricerca in Agricoltura e l’Analisi dell’Economia Agraria, Unità di Ricerca per l’OrticolturaMontanaso Lombardo, Italy

**Keywords:** *S. melongena*, chlorogenic acid, RACE, qRT-PCR, gene regulation, genome walking

## Abstract

Phenylpropanoids are major secondary metabolites in eggplant (*Solanum melongena*) fruits. Chlorogenic acid (CGA) accounts for 70–90% of total phenolics in flesh tissues, while anthocyanins are mainly present in the fruit skin. As a contribution to the understanding of the peculiar accumulation of these health-promoting metabolites in eggplant, we report on metabolite abundance, regulation of CGA and anthocyanin biosynthesis, and characterization of candidate CGA biosynthetic genes in *S. melongena*. Higher contents of CGA, Delphinidin 3-rutinoside, and rutin were found in eggplant fruits compared to other tissues, associated to an elevated transcript abundance of structural genes such as *PAL*, *HQT*, *DFR*, and *ANS*, suggesting that active *in situ* biosynthesis contributes to anthocyanin and CGA accumulation in fruit tissues. Putative orthologs of the two CGA biosynthetic genes *PAL* and *HQT*, as well as a variant of a *MYB1* transcription factor showing identity with group six MYBs, were isolated from an Occidental *S. melongena* traditional variety and demonstrated to differ from published sequences from Asiatic varieties. *In silico* analysis of the isolated *SmPAL1*, *SmHQT1*, *SmANS*, and *SmMyb1* promoters revealed the presence of several Myb regulatory elements for the biosynthetic genes and unique elements for the TF, suggesting its involvement in other physiological roles beside phenylpropanoid biosynthesis regulation. Transient overexpression in *Nicotiana benthamiana* leaves of *SmMyb1* and of a *C-*terminal *SmMyb1* truncated form (*SmMyb1Δ9*) resulted in anthocyanin accumulation only of *SmMyb1* agro-infiltrated leaves. A yeast two-hybrid assay confirmed the interaction of both *SmMyb1* and *SmMyb1Δ9* with an anthocyanin-related potato bHLH1 TF. Interestingly, a doubled amount of CGA was detected in both *SmMyb1* and *SmMyb1Δ9* agro-infiltrated leaves, thus suggesting that the N-terminal region of *SmMyb1* is sufficient to activate its synthesis. These data suggest that a deletion of the C-terminal region of *SmMyb1* does not limit its capability to regulate CGA accumulation, but impairs anthocyanin biosynthesis. To our knowledge, this is the first study reporting a functional elucidation of the role of the C-term conserved domain in MYB activator proteins.

## Introduction

Eggplant, also known as brinjal, is a berry-producing vegetable belonging to the large Solanaceae family and, similarly to other popular and important Solanaceous crop such as tomato, potato, and pepper, is cultivated across all continents. Eggplant is represented by three cultivated species, *Solanum macrocarpon* L. and *S. aethiopicum* L., which are indigenous to a vast area of Africa and are locally cultivated, and the worldwide cultivated *S. melongena* L., which was domesticated in multiple locations of the Asian continent (Knapp et al., 2013). Thus, opposite to the other widely cultivated Solanaceae, tomato, potato, and pepper, which are native of the New World (Fukuoka et al., 2010; Albert and Chang, 2014; Hirakawa et al., 2014), eggplant has a phylogenetic uniqueness, due to its exclusive Old World origin.

In the Solanaceae family, eggplant is the second most consumed fruit crop after tomato. Although generally considered as a “low-calorie vegetable,” the nutritional value of its fruits is comparable to most common vegetables, and they are also rich in important phytonutrients like phenolic compounds and flavonoids, many of which have antioxidant activities (Raigón et al., 2008), conferring to this vegetable a high nutritional value and extraordinary health-promoting effects (Stommel and Whitaker, 2003). As basic ingredient of the Eastern cuisine, it has been shown that daily eggplant dietary intake appears to be linked to a reduction of chronical disease risks (McCullough et al., 2002). In fact, eggplant has been used in traditional medicine; its tissue extracts have been considered useful for the treatment of asthma, bronchitis, cholera, and dysuria, beneficial in lowering blood cholesterol and showed also antimutagenic properties (Khan, 1979; Hinata, 1986; Kalloo, 1993; Collonnier et al., 2001; Kashyap et al., 2003).

Chlorogenic acid (CGA) is the main phenylpropanoid metabolite in the Solanaceae (Niggeweg et al., 2004). Growing interest for this molecule is due to its many beneficial properties for the treatment of various metabolic and cardiovascular diseases (Dos Santos et al., 2006; Cho et al., 2010; Plazas et al., 2013a). Moreover, CGA is highly stable at high temperatures, and its bioavailability in eggplant increases after cooking compared to the raw product (Lo Scalzo et al., 2010, 2016). CGA is accumulated in all plant tissues, reaching the highest amount in fruits, ranging from 75 to 90% of total phenolics. Other phenylpropanoid compounds include the purple and red anthocyanic pigments (D3R and Nasunin) and the flavonols, which are reported to be the major antioxidant constituents in eggplant fruit skin (Mennella et al., 2010). Along with CGA, anthocyanins and flavonols display considerable health-promoting effects due also to their ability to modulate mammalian cell signaling pathways (Meiers et al., 2001; Lamy et al., 2006). The three initial reactions of the phenylpropanoid pathway are catalyzed by phenylalanine ammonia lyase (PAL), cinnamate 4-hydroxylase (C4H) and 4-coumaroyl CoA ligase (4CL), to provide the high energy intermediate Coumaroyl-CoA ester. In eggplant, 4-Coumaroyl CoA is esterified with quinic acid by the hydroxycinnamoyl CoA-quinate transferase (HQT) enzyme to form CGA, and is also the substrate for the chalcone synthase (CHS) enzyme to form naringenin, the entry molecule of the flavonoid pathway (Vogt, 2010).

In several fruits and vegetables, such as apple, tomato, onion, and potato, skin and flesh tissues are often characterized by a distinct metabolite composition or content (Vrhovsek et al., 2004; Takos et al., 2006; Mintz-Oron et al., 2008; Stushnoff et al., 2010). This is also true for eggplant, whose phenylpropanoid profile differs between skin and fruit flesh, indicating that their degree of accumulation is tightly regulated (Mennella et al., 2010, 2012; Plazas et al., 2013b).

The production of phenylalanine-derived compounds in plants is mostly regulated by R2R3-MYB proteins, which are the largest class of secondary metabolism modulators (Stracke et al., 2007). A number of 222, 138, 118, 244 R2R3-MYB proteins have been reported in apple, *Arabidopsis thaliana*, grapevine, and soybean, respectively (Matus et al., 2008; Wilkins et al., 2009; Du et al., 2012; Katiyar et al., 2012; Cao et al., 2013), and more than a hundred seems to be present in eggplant (The Italian Eggplant Genome Consortium, unpublished).

Functional redundancy has been often reported for this class of MYB TFs, since several structurally related MYB TFs have been shown to activate identical gene subsets by interacting with the same *cis* elements in their gene promoters (Hartmann et al., 2005; Stracke et al., 2007). Differently, structural genes controlling the late steps of the anthocyanin biosynthetic pathway are regulated by a ternary transcriptional complex composed by members of the R2R3-MYB family (like, in *Arabidopsis*, Myb114, Myb111, PAP1, and PAP2), in combination with bHLH TFs (TT8, GL3, and EGL3) and WD40 repeat proteins such as TTG1 (Dare et al., 2008; Petroni and Tonelli, 2011; Laursen et al., 2015).

Albeit several MYB R2R3-TFs seem to regulate the same activation program, localization and expression studies indicate major differences in their spatial and temporal expression pattern, thus suggesting that their recruitment is indeed selective (Feller et al., 2011). Moreover, endogenous signals, such as cell or tissue specificity, as well as exogenous stimuli, operate to fine tuning this network, thus making phenylpropanoid pathway regulation extremely accurate (Hahlbrock et al., 2003; Wilkins et al., 2009). In this regard, it has been reported that heterologous expression of several MYB TFs induces the biosynthesis of phenylpropanoids in a species-specific manner (Docimo et al., 2013), even in closely related species. For example, the *A. thaliana* and tobacco flavonol regulator *AtMyb12*, when heterologously over-expressed in tomato plants, leads to the activation of off-target genes, determining an increase of CGA content, thus indicating that target genes transactivation might differ between different plant species (Luo et al., 2008).

Despite the phenylpropanoid pathway and its regulation, as well as the members of the MYBs-WRD40-bHLH complex, have been extensively studied in many plant species including Solanaceae (Spelt et al., 2000; Thorup et al., 2000; Pattanaik et al., 2010; Povero et al., 2011; D’Amelia et al., 2014; Kiferle et al., 2015; Montefiori et al., 2015) the peculiar high production of CGA in eggplant has been investigated to a lesser extent. Genetic studies pointed to the understanding of quantitative traits loci (QTL) affecting either CGA or anthocyanin content, in order to address breeding programs toward the improvement of these quality traits (Barchi et al., 2011; Plazas et al., 2013b; Gramazio et al., 2014). Gramazio et al. (2014) mapped candidate CGA biosynthetic genes on a interspecific map (*S. melongena* × *S. incanum*) on chromosomes E01, E03, E06, E07, and E09. More recently, a QTL study for the metabolic content of anti-nutritional and flavor and health-related metabolites performed on a intra-specific map of eggplant already described by Barchi et al. (2011, 2012) allowed the localization of two conserved major/minor QTLs for CGA on chromosomes E04 and E06 (Toppino et al., unpublished).

The recently published draft genome of the Asian eggplant cv. ‘Nakate-Shinkuro’ (Hirakawa et al., 2014) and a rich ESTs collection (Fukuoka et al., 2010) are providing valuable information on the accumulation of metabolites of interest for eggplant. Mining of the eggplant draft genome revealed a single HQT gene, but multiple copies of putative CH3 genes, belonging to the CYP family, and their comparison with tomato and potato genes suggested that the CGA biosynthetic pathway might have encountered a different evolution in eggplant compared to these two species, possibly to grant a higher metabolic rate (Fukuoka et al., 2010; Hirakawa et al., 2014). Besides, two *Myb-like* genes were also identified, supposedly involved in controlling anthocyanin accumulation in the flower (Hirakawa et al., 2014), while the *SmMYB1* gene, isolated from a cultivar with purple fruits, was able to drive anthocyanin accumulation in over-expressing shoots (Zhang et al., 2014). A few more studies addressed the biosynthesis of flavonoids or the regulative mechanisms responsible for the high presence and accumulation of anthocyanins in eggplant, and indicate that domestication of *S. melongena* might have affected the accumulation of phenolic compounds (Meyer et al., 2015), as well as altered the regulation of some anthocyanin target genes (Doganlar et al., 2002). Hence, the presence of multiple copies of structural genes and the redundancy of different regulatory proteins suggest that evolutionary mechanisms affected qualitative and quantitative accumulation of CGA and anthocyanins.

Further studies on metabolite distribution and relevant gene expression along with the isolation and characterization of structural and regulatory genes are needed to better explain the peculiar accumulation of metabolites in eggplant.

To this aim, we investigated phenylpropanoid accumulation in several tissues and organs of the Occidental eggplant cv. ‘Lunga Napoletana’ by LC–MS analysis, and characterized the spatial and temporal expression of the relative structural and potential regulatory genes by qRT-PCR. We report here our independent isolation of S*mPAL* and *SmHQT* key genes for CGA biosynthesis and of a genetic variant of the recently isolated MYB TF *SmMyb1* (Zhang et al., 2014). Although pathway genes are fully represented in the eggplant draft genome^1^, in this study we provide further indication of the genetic diversity between *S. melongena* varieties. To expand our understanding of the regulatory mechanisms underlying phenylpropanoid accumulation, we also isolated *SmANS* (Anthocyanidin synthase) and *SmMyb1* promoter sequences, whose *in silico* analysis for *cis*-acting elements highlighted common regulatory motifs, suggesting a possible coordinated regulation. On the contrary, comparison with other anthocyanins and phenolic acids-related TFs revealed distinctive regulatory motifs of the isolated eggplant *SmMyb1* promoter.

Finally, to assess the function of *SmMyb1*, both the entire coding sequence and a truncated C-terminal form were transiently over expressed in tobacco leaves and their effects were evaluated by molecular and biochemical analysis. In addition, the ability of *SmMyb1* to interact with a bHLH partner was assessed by yeast two hybrid assay. To our knowledge this is the first time that the function of the C-terminal domain in MYB activator proteins is reported.

Our results indicate that the regulatory function of the isolated genetic variant of *SmMyb1* is not limited to the activation of anthocyanin biosynthesis but might also have a role in regulation of CGA accumulation

## Materials and Methods

### Plant Material

*Solanum melongena* cultivar “Lunga Napoletana” with purple-black oblong fruits, was cultivated in the greenhouse of the CNR-IBBR, UOS Portici, (Italy). Samples were all harvested when fruits reached a commercially ripe stage (Mennella et al., 2012). Flowers, leaves in two stages (young and mature), stem, roots and fruits (skin and flesh) were simultaneously collected for biochemical and molecular analyses. Samples from three different plants were frozen in liquid nitrogen and stored for further molecular and biochemical analysis.

*Nicotiana benthamiana* plants for transient transformation assays were grown in growth chamber of CNR-IBBR, UOS Portici, (Italy) at the temperature of 22°C with a photoperiod of 16 h light/8 h dark. After 1 month, youngest leaves were used for transient assays. Leaf samples were collected in liquid nitrogen and stored at –80°C for further biochemical and molecular analyses.

### LC–MS Analysis of Phenylpropanoids

Anthocyanins, flavonoids and CGA were analyzed by mass spectrometry in different tissues and organs of the *S. melongena* cultivar “Lunga Napoletana” and in *N. benthamiana* leaves. Metabolic analysis of *N. benthamiana* agro-infiltrated leaves was carried out on three independent replicates collected for each infiltration.

Phenylpropanoids were extracted according to the following protocol. Briefly, 5 and 25 mg of lyophilized samples, respectively, for eggplant and *N. benthamiana*, were extracted in 1.5 ml of 75% (v/v) methanol containing 0.05% (v/v) trifluoroacetic acid (TFA). After homogenization, the samples were stirred for 40 min and centrifuged at 19,000 × *g* for 10 min. The extracts were filtered through 0.2 μm polytetrafluoroethylene filters. For each tissue and/or genotype, three biological replicates (each in two technical replicates) were prepared. All the extracts were analyzed through reversed phase liquid chromatography coupled to a photodiode array detector and to an ion trap mass spectrometry (LC–PDA–MS) system. Such a system consisted of an ultra-performance liquid chromatography (UPLC) DIONEX Ultimate 3000 model coupled to a LTQ XL mass spectrometer (Thermo Fisher Scientific). A 5 μl aliquot of sample was injected on a Luna C18 (100 mm × 2.0 mm, 2.5 μm particle size) column equipped with a Security Guard column (3.0 mm × 4.0 mm) from Phenomenex. The separations were carried out using a binary gradient of ultrapure water (A) and acetonitrile (B), both acidified with 0.1% (v/v) formic acid, with a flow rate of 0.22 ml/min.

The initial solvent composition consisted of 95% (v/v) of A and 5% (v/v) of B; increased linearly to 25% A and 75% B in 25 min and maintained for 1 min; returned to 95% of A in 1 min. The column was equilibrated to 95% A and 5% B for 11 min before the next injection. The analysis lasted for 38 min and the column temperature was set to 40°C. Mass spectra were obtained in positive ion mode over the range *m/z* 70–1,400. The capillary voltages were set at 9.95 V and the source temperature was 34°C. Quantitative determination of compounds was conducted by comparison with dose–response curves based on *m/z* data from authentic, distinct and appropriately diluted standard solutions of D3R (Polyphenols Laboratories AS, Sandnes, Norway), CGA and rutin (Sigma–Aldrich, St. Louis, MO, USA). Xcalibur software (Thermo Fisher Scientific) was used to control all instruments and for data acquisition and data analysis.

### RNA Isolation and qRT-PCR

Total RNA was extracted from 100 mg of eggplant tissues and organs using an RNAsy kit (Qiagen, Valencia, CA, USA). Using a Super ScriptII^TM^ kit (Life Technologies, Carlsbad, CA, USA), first-strand cDNA was synthesized by reverse transcription (RT) with oligo-dT primers following the manufacturer’s instructions. Gene expression was analyzed using qRT-PCR, which was performed using an ABI7900 HT (Life Technologies, Carlsbad, CA, USA). To amplify the gene fragments, 1 μL of 1:25 diluted cDNA was used as a template in a 20 μL PCR reaction with 10 μL SYBR Green, and 0.4 μM of each primer. The PCR reaction was conducted as follows: 50°C for 2 min, followed by incubation for 30 s at 95°C and denaturation for 15 s at 95°C, annealing for 20 s at 60°C, and 40 cycles of elongation at 72°C for 20 s. The analysis was done on three biological replicates and in technical triplicate. A relative standard curve for each gene was developed using fourfold serial diluted cDNA and included in all runs to relate to quantitative data. PCR efficiency of primer pairs was optimized to be between 79 and 97% with *R*^2^-values of 0.985. PCR product melting curves were analyzed for the presence of a single peak, showing that only one PCR product is formed. PCR products were cloned and sequenced to verify that all primer pairs targeted the desired RNA. Adenine phosphoribosyltransferase (*APRT*) was used as internal reference gene since its expression was found stable in all the analyzed tissues as also reported by Gantasala et al. (2013). Results were analyzed using the ^ΔΔ^Ct method (Pfaﬄ, 2001, 2004) and reported as relative expression levels, compared to young leaves as internal calibrator.

Expression analysis on *N. benthamiana* was performed on RNA extracted from 5 days post agro-infiltration leaves. The results were expressed in the form of relative expression through the ^ΔΔ^Ct method, by using tobacco wild type leaves as internal calibrator tissue. Normalization was performed by using α*-Tubulin* as housekeeping gene, since its expression was stable as reported by Pattanaik et al. (2010).

A list of the analyzed genes, accession numbers, and primer sequences can be found in Supplementary Table S1. For tobacco qPCR primers, the sequences used in this study are identical to primer pair sequences reported by Pattanaik et al. (2010).

### *SmPAL, SmHQT*, and *SmMyb1* Genes Isolation and Cloning

Gene isolation from the Occidental traditional eggplant cv. ‘Lunga Napoletana’ was initially attempted by using degenerate primers designed on *PAL*, *HQT*, and *Myb* nucleotidic sequences from other Solanaceae, which however, amplified several unspecific fragments. Therefore, 5′3′ RACE strategy was used for gene isolation. Sequences available for *PAL* and *HQT* from Solanaceae were used to BLAST search orthologs in a *S. melongena* ESTs collection (Fukuoka et al., 2010). Gene specific primers were designed on ESTs FS058603.1 and FS083932.1 for *PAL* and *HQT*, respectively. For *Myb1* isolation, primers designed on the *S. melongena* FS084890 EST were used for 3′–5′ end RACE. Total RNA extracted from *S. melongena* fruit tissues was used as a template to amplify the *SmPAL*, *SmHQT*, and *SmMyb1*cDNAs. Both 3′–RACE (3′-RACE System, Life Technologies, Carlsbad, CA, USA) and 5′-RACE (Smart Race Kit, Clontech, Mountain View, CA, USA) were performed, following the manufacturers’ instructions. Two groups of two gene-specific primers, 3′GSP1, 3′GSP2, and 5′GSP1, 5′GSP2, (Supplementary Table S1) were used for 3′-RACE and 5′-RACE, for S*mPAL* and *SmHQT*, respectively. Touchdown-PCR reactions were performed as follows: 3 min pre-denaturation at 94°C, followed by 94°C for 30 s, 68°C for 30 s, and 72°C for 1 min in the first cycle, then decreasing the annealing temperature by 1°C/cycle for 11 cycles, followed by 94°C for 30 s, 57°C for 30 s, and 72°C for 1 min for 19 cycles and ending with 7 min of elongation at 72°C. Amplified cDNA fragments were ligated to the TOPO TA vector (Life Technologies, Carlsbad, CA, USA) following the manufacturer’s instructions. Recombinant bacteria growing on kanamycin selective media were screened and verified by PCR. All sequences were confirmed by DNA sequencing (Primm s.r.l. laboratories, Milan, Italy^2^).

Genomic DNA was extracted from eggplant leaves using the “DNAsy Plant mini kit” (Qiagen, Valencia, CA, USA). *SmMYB1* was amplified by PCR starting from genomic DNA isolated from eggplant leaves using *Phusion* DNA polymerase (Thermo Fisher Scientific, Waltham, Ma, USA) and specific primers (Supplementary Table S1). The amplified *SmMYB1* was cloned into TOPO-TA vectors and verified by sequencing as above reported.

### Bioinformatics and Statistical Analyses

The ORF finder program of Vector NTII was used to search for open reading frames in the putative full-length cDNAs of *S. melongena PAL* (KT259041), *HQT (*KT259042), and *Myb1 (*KT259043). The fundamental properties and structural features of the proteins were analyzed via ScanProsite^3^ Alignments of multiple amino acid sequences were carried out using ClustalW^4^ Phylogenetic trees of the *Sm*PAL, *Sm*HQT, and *Sm*MYB1 proteins were produced by Neighbor Joining matrix (Saitou and Nei, 1987) with 1,000 bootstrap trials using MEGA6 (Tamura et al., 2013). The evolutionary distances were computed using the p-distance method and are in the units of the number of amino acid differences per site (Nei and Kumar, 2000).

Analysis of variance (ANOVA) on qPCR ^Δ^Ct data was carried out using SigmaPlot version 12.0, from Systat Software Inc., San Jose, CA, USA^5^ Duncan’s test was performed to compare mean values. Pearson product moment correlation coefficients (*r*-values) were calculated by Systat Software using the means of metabolite concentrations or relative gene expression values.

### Promoter Cloning and Regulatory Elements Analysis

Promoter sequences for the *SmANS* and *SmMyb1* genes of the cv. ‘Lunga Napoletana’ were amplified by the Genome walking strategy (Clontech, Mountain View, CA, USA) by using gene specific primers (Supplementary Table S1) designed in order to amplify the 5′UTR region. Promoter regions longer than 1 Kb were isolated, cloned into TOPO-TA vectors (Life Technologies, Carlsbad, CA, USA) and sequenced. Putative promoter sequences for *SmPAL* and *SmHQT* were also obtained by Genome Walking, but sequence mining of the genome sequenced by the Italian Eggplant Consortium revealed that they in fact belonged to another *PAL* isoform and to a putative *HCT* highly similar to *S. tuberosum* (personal communication). Since the *ANS* and *MYB1* upstream sequences isolated from ‘Lunga Napoletana’ (this work) were found identical to the sequences from the genome of the Italian Eggplant Genome Consortium, we used the latter *PAL* and *HQT* promoters for further studies, after verifying sequence identity. Promoter sequences corresponding to *S. lycopersicum ANT1*, *S. tuberosum AN1*, *S. tuberosum CAI*, and *Vitis vinifera* cultivar Pinot Noir *VvMybA1* were retrieved from the respective publically available genomic sources. Analysis of *cis*-regulatory elements was performed through the Genomatix platform^6^

### *SmMyb1* Transient Expression in *Nicotiana benthamiana*

*SmMyb1* cds was *Pfu* amplified with primers designed for pENTR-D-TOPO cloning vector (Life Technologies, Carlsbad, CA, USA). *SmMyb1* gene from the entry clone was cloned in the 35SCaMV expression cassette of pGWB411 (Nakagawa et al., 2009) using the Gateway recombination technology (Invitrogen, Carlsbad, CA, USA). Spectinomycin positive colonies were sequenced and used to transform *Agrobacterium tumefaciens* LBA4404.

*Nicotiana benthamiana* plants were grown until they had six leaves and the youngest leaves over 1 cm long were infiltrated with *A. tumefaciens* LBA4404. Bacteria were cultured on Lennox agar (Life Technologies, Carlsbad, CA, USA) supplemented with 50 μg ml^-1^ kanamycin (Sigma–Aldrich, St. Louis, MO, USA) and incubated at 28°C. A 10 μl loop of confluent bacteria were re-suspended in 10 ml of infiltration media (10 mM MgCl_2_, 0.5 μM acetosyringone), to an OD_600_ of 0.3, and incubated at room temperature without shaking for 2 h before infiltration. Infiltrations were performed according to the method of Voinnet et al. (2003). Approximately 300 μl of the *Agrobacterium* suspension were infiltrated into a young leaf of *N. benthamiana* and transient expression was assayed 5 days post inoculation.

### Yeast Two-Hybrid Assay

For yeast two-hybrid experiments, the prey plasmid pGADT7 (Clontech, Mountain View, CA, USA) was used. The full-length coding sequence of *SmMyb1* and a truncated form lacking the last nine amino acids (*SmMyb1Δ9*) were PCR amplified and cloned in frame into pGADT7 between *Eco*RI and *Xho*I restriction sites. Plasmids were sequenced to rule out PCR-induced mutations. The bait plasmid StbHLH1pGBKT7 was previously described (D’Amelia et al., 2014). The bait and prey plasmids were transformed into the yeast strain AH109 (Clontech, Mountain View, CA, USA) using the Lithium acetate/Polyethylene glycol method (Bai and Elledge, 1997). The self-activation test was performed prior to the testing of combinations of interest. In particular an equal amount of cells transformed with the prey plasmid pGADT7 containing *SmMyb1* or *SmMyb1Δ9* was spotted on medium lacking leucine and medium lacking adenine, histidine, leucine. The same was done for the bait plasmid StbHLH1 pGBKT7, that was grown on medium lacking tryptophan and medium lacking adenine, histidine, and tryptophan. After verifying that the bait and prey plasmids when transformed alone conferred ability to grow on tryptophan or leucine, respectively, indicating presence of the plasmid, but not on media lacking three amino acids, which would have indicated self-activation, co-transformations to verify interactions were performed. Transformed colonies containing bait and prey plasmids were selected on synthetic drop-out medium lacking leucine and tryptophan (–W/–L). Co-transformants were grown overnight in liquid culture lacking leucine and tryptophan (–W/–L). For the interaction between bait and prey, an equal amount of cells was spotted on medium lacking adenine, histidine, leucine and tryptophan (–W/–L/–H/–A). Positive and negative controls were also performed as indicated in the legend of **Figure 6**.

### Accession Numbers

The cloned sequences for *SmPAL* (KT259041), *SmHQT* (KT259042), *SmMyb1* (KT259043) CDS, *SmMyb1* genomic and promoter sequence (KT727965) and *ANS* promoter (KT727965) sequences were submitted to the GenBank/EMBL database. Promoter sequences for *SmPAL* (KT591485) and *SmHQT* (KT591484) were kindly provided by the Italian Eggplant Genome Consortium. The genomic localization of the analyzed promoters were Chr10: 64468200…64466701 for *SlANT1*, 219865…218366 for *StAN1*; Ch9: 163301…161802 for *StCAI*; Ch2: 14242103…14240604 for *VvMybA1*. Sequences used for phylogenetic analyses are reported in **Figure 3** and Supplementary Figures S1–S3.

## Results

### Phenylpropanoid Content in *S. melongena* Cultivar “Lunga Napoletana”

The patterns of accumulation of the major phenylpropanoid metabolites of *S. melongena*, namely the anthocyanin D3R, the phenolic acid CGA and the flavonoid rutin were investigated in several tissues and organs of the eggplant cultivar “Lunga Napoletana,” namely two leaf stages (young and mature), stems, flowers, roots, and fruit skin and flesh.

**FIGURE 1:**
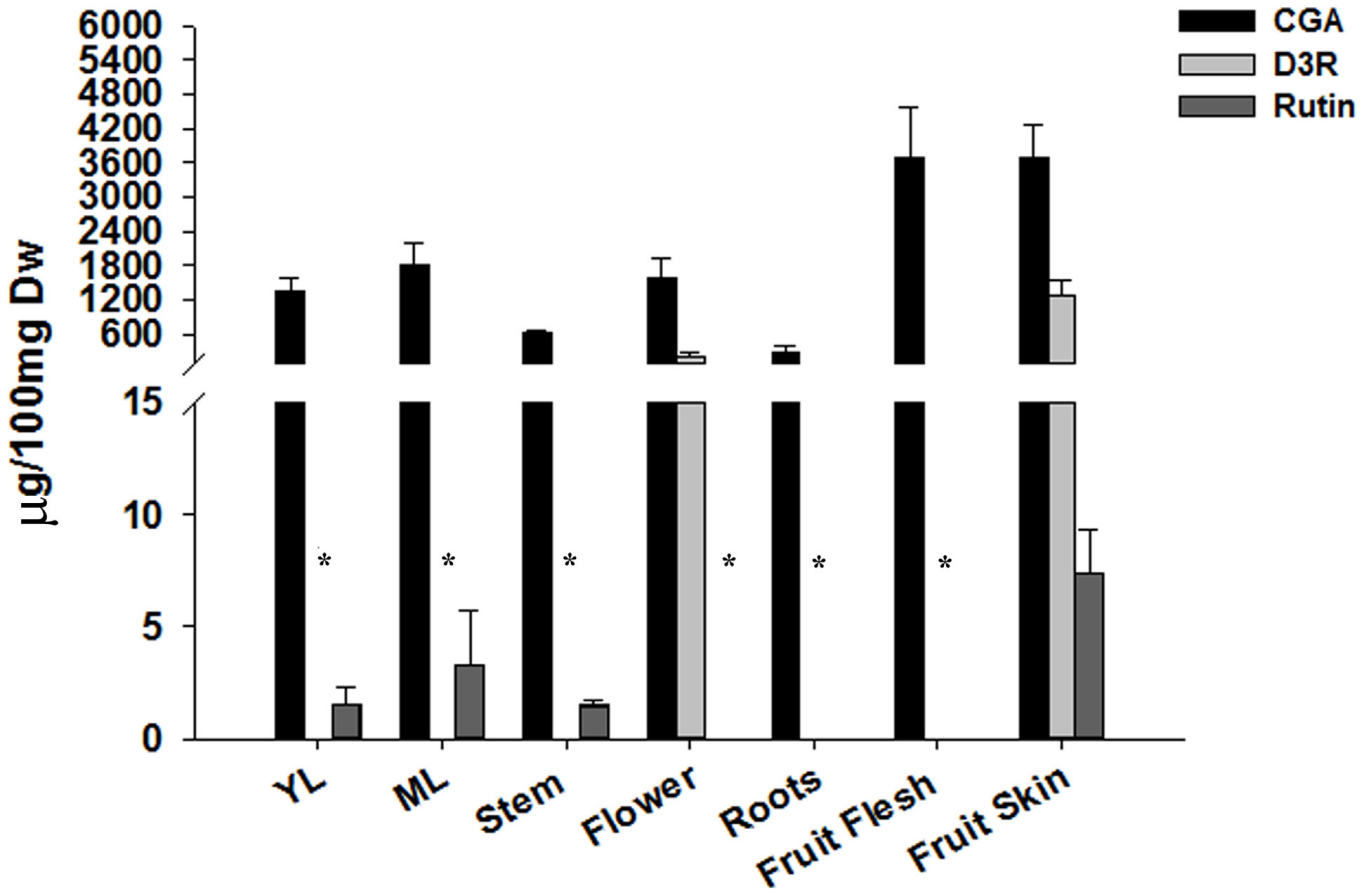
**LC–MS analysis of phenylpropanoids.** Chlorogenic acid, delphinidin 3-rutinoside, rutin were extracted from 100 mg of lyophilized tissue in 75% MeOH and quantified by LC–MS. Values are expressed as means ± SD (*n* = 3). ^∗^Compounds were not detected.

As expected, the D3R content mirrored anthocyanic pigmentation in all the considered tissues. A concentration of about 200 μg/100 mg dw was detected in flowers, while a six times higher amount (∼1200 μg/100 mg dw) was measured in the eggplant fruit skin (**Figure 1**). CGA was detected in all the tissues, and its amount ranged from 1300 to 1800 μg/100 mg dw in leaves and flowers to more than 3000 μg/100 mg dw in fruits. The lowest content was detected in stems and roots, with 600 and 250 μg/100 mg dw, respectively (**Figure 1**).

The flavonoid rutin was detected only in leaves at both stages, stems and fruit skin and its amount in the green tissues ranged from 1 μg in stems and young leaves to 3 μg/100 mg dw in mature leaves, while about a three times higher content was detected in the fruit skin.

Overall, the fruits showed the highest content of CGA, D3R, and rutin.

### Expression Analysis of Phenylpropanoid Biosynthetic Genes

**FIGURE 2:**
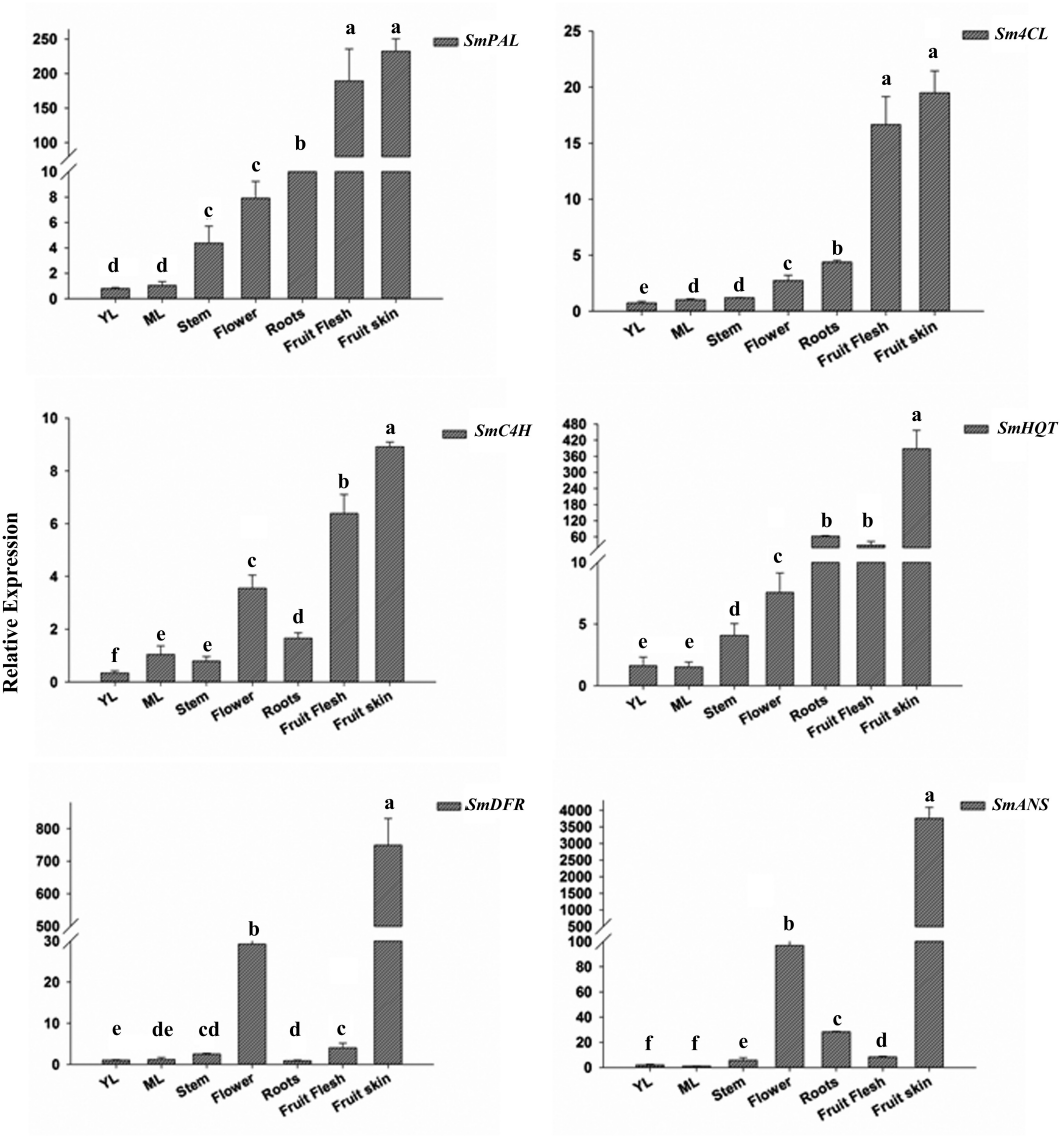
**Relative transcript levels of *SmPAL, SmC4H, Sm4CL, SmHQT, SmDFR*, and *SmANS* in various *Solanum melongena* organs and at two leaf development stages (YL, young leaves and ML, mature leaves).** The results were analyzed using the ^ΔΔ^Ct method and presented as fold changes compared with the young leaves, used as internal calibrator. Data are reported as means ± SD. Means denoted by the same letter did not differ significantly at *p* ≤ 0.05 according to Duncan’s multiple range test.

Quantitative expression analyses were performed in the same tissues sampled for accumulation of metabolites. The transcript abundance of both the early genes, i.e., *PAL*, *C4H*, and *4CL*, and the late genes of the phenylpropanoid pathway encoding for enzymatic steps leading to CGA, D3R, and rutin biosynthesis, namely hydroxycinnamoyl-CoA quinate transferase (*HQT*), dehydroflavonol reductase (*DFR*), and anthocyanidin synthase (*ANS)* were analyzed (**Figure 2**).

*PAL*, *C4H*, and *4CL* transcripts were detected in all the tissues, with a lower transcript abundance being observed in green tissues (young and mature leaves and stem) than in flowers, roots and fruits, where the expression levels were overall higher. Expression levels were notably high in fruits, where *PAL* transcripts were 1 to 2 orders of magnitude more abundant than in the other tissues. Expression of *HQT*, the key biosynthetic gene in CGA formation, was strongest in both fruit skin and flesh, while showing low expression levels in the other tissues.

Anthocyanin and flavonoid common biosynthetic genes for D3R and rutin formation were also examined. *DFR* and *ANS* transcripts showed a similar pattern of accumulation, with higher expression levels detected in anthocyanin-pigmented tissues, i.e., in flowers and fruit skin. Interestingly, the expression of the two genes was almost 25 and 35 times higher in fruit skin than in flowers, respectively. On the contrary, transcripts levels were significantly lower in non-anthocyanic pigmented tissues.

### *PAL* and *HQT* Genes Isolation

Our biochemical and gene expression results indicated that a high accumulation of CGA mainly occurs in fruits, due to the up-regulation of its biosynthetic genes at the transcriptional level. Therefore, isolation of *PAL* and *HQT* encoding genes was achieved through RACE PCR starting from fruit tissues mRNA. Since at the time of the experiments the eggplant draft genome was not available yet, we used conserved *PAL* and *HQT* sequences from tomato and potato to mine an eggplant ESTs collection through BlastN. Gene specific primers designed on the two *S. melongena* ESTs FS058603.1 and FS083932.1, corresponding to putative *SmPAL* and *SmHQT*-encoding sequences, respectively, amplified single products by 5′ 3′ RACE PCR. Regarding *SmPAL*, a 2712 bp fragment was cloned and confirmed by sequencing to contain a full length ORF of 2430 bp, encoding for a 724 aa protein of 78.7 kD molecular mass and isoelectric point at 6.4 pH. The sequence isolated from ‘Lunga Napoletana’ was blasted in the eggplant draft genome, and several partial sequences were found. Sequence comparison showed a similarity of 89% with Sme2.5_03336.1_g00008.1, an eggplant sequence annotated as PAL1 (Supplementary Figure S1A). On the contrary, higher similarity was found with orthologous *PAL* members from other Solanaceae, 93% with *Capsicum annuum* and *Solanum tuberosum* and 92% with *Solanum lycopersicum* (Supplementary Figure S1A). Prosite scan revealed that *S. melongena* PAL, similarly to all the other PAL proteins, possesses the typical features of an Histidine Lyase protein with the conserved active site (GTITASGDLVPLSYIA), including the Ala-Ser-Gly motif at position 206–208, which autocatalytically forms the methylidine-4h-imidazol-4-one (MIO) prosthetic group (MacDonald and D’Cunha, 2007) by cyclization and dehydration. Moreover, the residues involved in the modulation of PAL activities, i.e., Gly501 in the active site pocket, and Thr556 in the post transcriptional phosphorylation site are also conserved, thus suggesting that *SmPAL* is a functionally active protein.

Sequence analysis of the cloned 5′ and 3′ RACE-PCR *SmHQT* fragment identified a 1694 bp full length sequence containing an ORF of 1284 bp, encoding for a putative protein of 428 aa, with 47.6 kD molecular mass and isoelectric point at 6.4 pH. The predicted eggplant HQT from cv. ‘Lunga Napoletana’ was blasted in the eggplant draft genome, and a 96% similarity was found with Sme2.5_00673.1_g00011.1, an HQT-like gene lacking the N-terminal portion. Then *Sm*HQT was aligned to other dicot members encoding HQT protein, and a high degree of similarity was found with other Solanaceous HQTs (89% to *Solanum lycopersicum*, 88% to *Solanum tuberosum*, and 87% to *Nicotiana tabacum*). *Sm*HQT possesses the characteristic HTLSD peptide of acyltransferase proteins from position 153, corresponding to the conserved sequence motif HXXXDG, and the DFGWG block from position 383, observed in other plant acyltransferases belonging to the BAHD family (St-Pierre and De Luca, 2000; D’Auria et al., 2002; Supplementary Figure S2A).

The results for *S. melongena* PAL and HQT protein sequences were used to construct phylogenetic trees using the Neighbor Joining method and illustrate their evolutionary relationships with respect to the related enzymes from other plants. Notably, eggplant proteins encoded by the *SmPAL* and *SmHQT* genes isolated in this study cluster within the same clade with other Solanaceae (tomato, potato, and tobacco) characterized enzymes, as shown in Supplementary Figures S1B and S2B.

### *Myb1* Isolation and Characterization

In search for a MYB TF responsible for the activation of the phenylpropanoid pathway in eggplant, the eggplant ESTs database was searched by BLAST with the CDS of *S. tuberosum* Chlorogenate inducer (CAI; EU310399), since neither the sequence of the eggplant *SmMyb* TF by Zhang et al. (2014) nor those of the eggplant draft genome were publically available yet. Primers for qRT-PCR were designed on the identified *S. melongena* FS084890 EST and adapted for 5′3′ end RACE PCR of eggplant leaf RNA. Cloning and sequencing of the amplified fragments revealed a full length cDNA of 1197 bp containing a 771 bp ORF, which from BLAST analysis was found homologous to a sequence recently isolated from fruits of the Chinese eggplant cv. ‘Zi Chang’ and recorded as *MYB1* (KF727476; Zhang et al., 2014). The *S. melongena* “Lunga Napoletana” *Myb1* variant differed from this sequence for the presence of four SNPs at positions 260, 675, 678, and 737, determining non-synonymous amino acid transitions in position 87 from Aspartate to Glycine and in position 246 from Serine to Phenylalanine. BLAST analysis of this MYB sequence in the draft eggplant genome demonstrated a 98% similarity at the nucleotidic level with the sequence Sme2.5_05099.1_g00002.1, annotated as *ANT1*. This eggplant isoform also showed four SNPs, which, however, did not result in amino acid transitions, and therefore encoded a MYB protein identical to the one encoded by our *SmMyb* sequence (Supplementary Figure S3).

**FIGURE 3:**
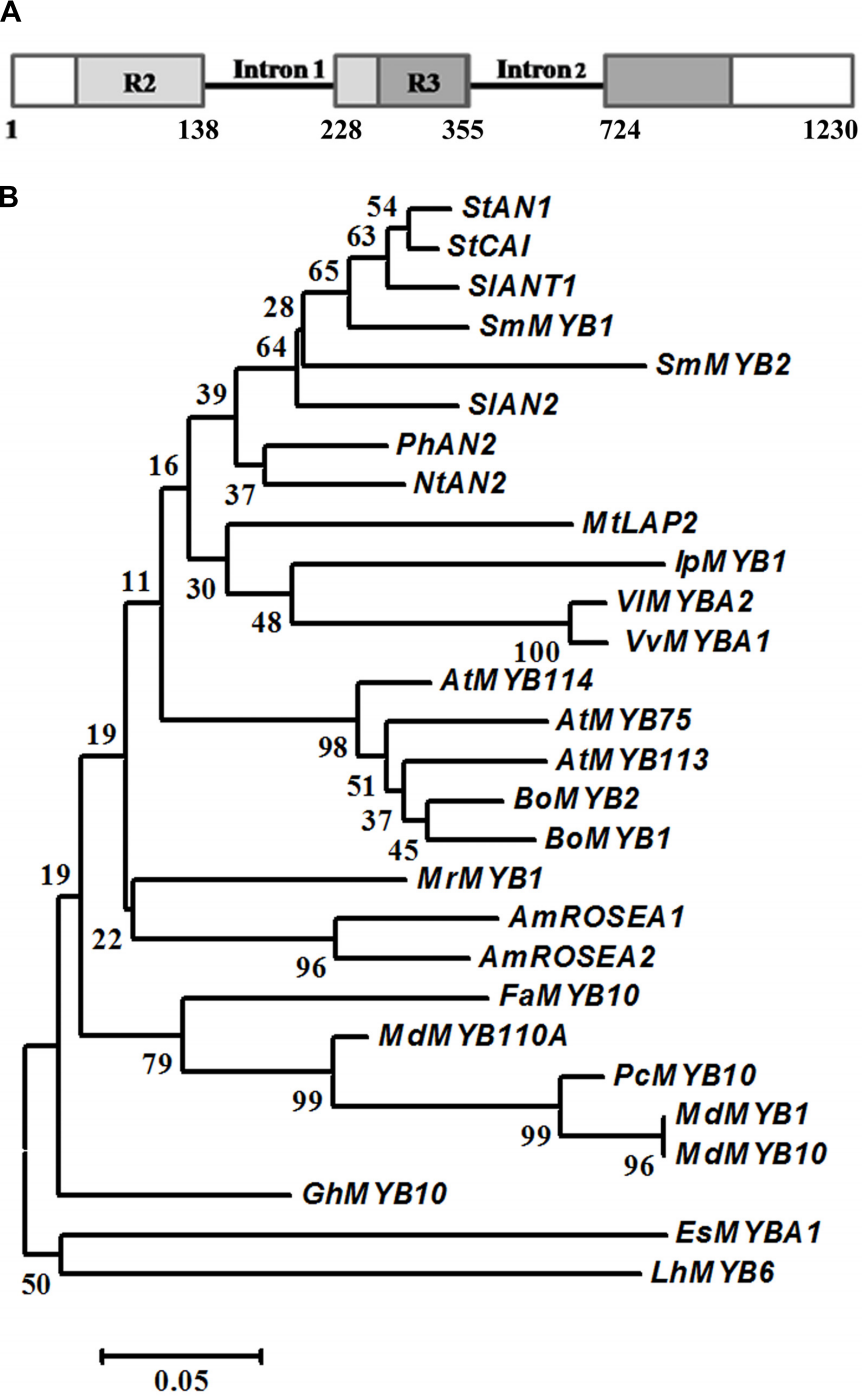
**Gene structure and phylogenetic analysis of *S. melongena Myb1*. (A)** Representation of *SmMyb1* genomic sequence (KT727965) and cDNA (KT259043). Light and dark gray boxes indicate the R2R3 domain in the *SmMyb1* coding sequence, solid black line indicates the intronic regions. **(B)** R2R3-MYB proteins from other species were aligned using Clustal X, and the evolutionary history was inferred using the Neighbor-Joining method. The optimal tree with the sum of branch length = 2.20562095 is shown. The percentage of replicate trees in which the associated taxa clustered together in the bootstrap test (1000 replicates) are shown next to the branches. The tree is drawn to scale, with branch lengths in the same units as those of the evolutionary distances used to infer the phylogenetic tree. The evolutionary distances were computed using the p-distance method and are in the units of the number of amino acid differences per site. The analysis involved 29 amino acid sequences. All positions containing gaps and missing data were eliminated. There were a total of 94 positions in the final dataset. Evolutionary analyses were conducted in MEGA6. Protein sequences used for the phylogenetic tree have the following accession numbers: *Malus domestica* MdMYB1 (ADQ27443.1); *Malus domestica* MdMYB10 (ACQ45201.1); *Malus domestica* MdMYB110a (AFC88038.1); *Arabidopsis thaliana* AtMYB75 (AEE33419.1); *Arabidopsis thaliana* AtMYB113 (NP_176811.1); *Arabidopsis thaliana* AtMYB114 (AEE34502.1); *Brassica oleracea* var. *botrytis* BoMYB2 (ADP76651.1); *Morella rubra* MrMYB1 (ADG21957.1); i GhMYB10 (CAD87010.1); *Vitis vinifera* VvMYBA1 (BAD18977.1); *Vitis vinifera* VvMYBA2 (BAC07540.1); *Ipomoea purpurea* IpMYB1 (BAE94388.1); *Nicotiana tabacum* NtAN2 (ACO52470.1); *Solanum lycopersicum* SlANT1 (AAQ55181.1); *Solanum lycopersicum* SlAN2 (FJ705333.1); *Antirrhinum majus* AmROSEA2 (ABB83827.1); *Antirrhinum majus* AmROSEA1 (ABB83826.1); *Solanum tuberosum* StCAI (ABY40370.1); *Solanum tuberosum* StAN1 (AGC31676.1); *Fragaria x ananassa* FaMYB (ABX79947); *Epimedium sagittatum* EsMYBA1 (AGT39059.1); *Lilium hybrid division I* LhMYB6 (BAJ05399.1); *Medicago truncatula* MtLAP2 (ACN79539.1); *Pyrus communis* PcMYB10 (ABX71487.1); *Solanum melongena* SmMYB2 (AIP93874); *Solanum melongena* SmMYB1 (KT259043).

Sequence comparison between the cDNA and the genomic sequence of *SmMyb1*, isolated from genomic DNA (KT727965) with specific primers designed on the start and stop codons of the cDNA, revealed that *SmMyb1* contains two introns, located in the R2R3 domain (**Figure 3A**), as reported for other *Myb* genes (Pattanaik et al., 2010).

Alignment of the encoded MYB1 protein of 258 aa with 12 R2R3-MYB proteins belonging to clade 6 (Liu et al., 2015) and known as anthocyanin and phenolic acids MYB regulators demonstrated high sequence homology in the R2R3 domain (Supplementary Figure S3), while less sequence homology is shared in the C-terminal region of all the sequences. *Sm*MYB1 shares 72 and 71% amino acid identity with *S. lycopersicum* ANT1 and *S. tuberosum* CAI, respectively, while 66, 56, and 47% homology is shared with *S. tuberosum* AN2, *V. vinifera* MYBA1 and *Petunia hybrida* AN2, respectively. A phylogenetic analysis was performed on the alignment of 28 R2R3-MYB protein sequences and the evolutionary history was inferred by using the Neighbor Joining method. The analysis included R2R3-MYB proteins involved not only in the activation of the anthocyanin pathway but also in the regulation of phenolic compounds.

Interestingly, *Sm*MYB1 clusters more closely to ANT1 from *S. lycopersicum* as well as with AN1 from potato thus suggesting that it may be the homologous protein in *S. melongena*. Noteworthily, *Sm*MYB1 clusters also with CaiMYB protein from potato (**Figure 3B**).

### Myb1 Expression Analysis in *S. melongena*

**FIGURE 4:**
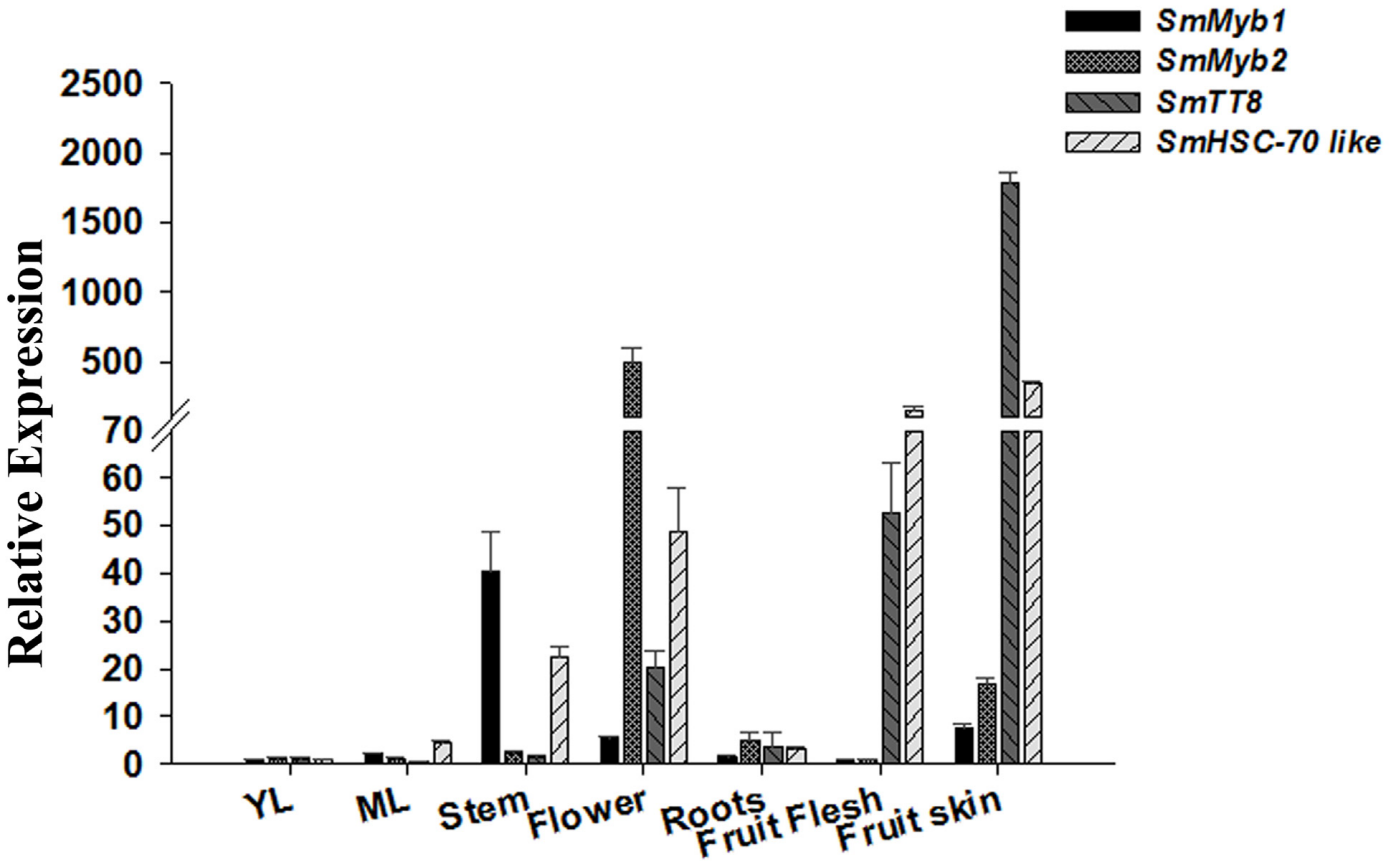
**Relative transcript levels of *SmMyb1*, *SmMyb2*, *SmTT8*, and *SmHSC-70-2-like* in various *S. melongena* organs and at two leaf development stages (YL, young leaves and ML, mature leaves).** The results were analyzed using the ^ΔΔ^Ct method and presented as fold changes compared with the young leaves, used as internal calibrator. Data are reported as means ± SD.

In order to further investigate the function of *SmMyb1* as putative regulator of phenylpropanoid accumulation, we performed a qPCR expression analysis of the distribution of *Myb1* transcripts in different *S. melongena* organs and tissues along with other three genes putatively involved in anthocyanin biosynthesis, namely *SmMyb2*, whose sequence mostly resembles the AN2 gene from Solanaceae (Supplementary Figure S4), *SmTT8* (KT591486), a putative homolog of the bHLH TF encoding gene *AtTT8* involved in anthocyanin regulation, and the heat shock cognate 70 protein 2 (*SmHSC70-2-like*, KT591487), which was previously found associated with a *S. melongena* QTL for anthocyanin accumulation mapping on chromosome 10 (Barchi et al., 2012). As shown in **Figure 4**, *SmMyb1* is expressed in all the tissues at a relatively low level except in stems. In the fruit flesh, both *SmMyb1* and *SmMyb2* show a low expression level, while *SmTT8* and *SmHSC70-2-like* are highly expressed. In addition, *SmMyb2* resulted to be induced at the highest level in flowers while *SmTT8* is mostly induced in the fruit skin. Interestingly, a higher *SmMyb2*, *SmTT8*, and *SmHSC70-2-like* transcript accumulation was observed in pigmented tissues, thus suggesting a correlation between the expression level of these regulatory genes and the accumulation of D3R (**Figures 1–4**).

### Isolation and *In Silico* Analysis of Phenylpropanoid Biosynthetic Genes and TFs Regulator Promoters

The coordinated expression of the CGA biosynthetic genes in fruit tissues as well as the high expression of late anthocyanin biosynthetic genes in the fruit skin, suggested that in *S. melongena*, as in other Solanaceae fruits, fruit skin and flesh are characterized by different metabolic processes and regulation (Jung et al., 2009). In order to investigate whether CGA and anthocyanin biosynthesis might be differentially regulated in eggplant, *PAL*, *HQT* and *ANS* gene promoters were *in silico* scanned for regulatory elements. MatInspector analysis (Cartharius et al., 2005) of 5′ upstream regions of 1546 bp for *SmPAL*, 1500 bp for *SmHQT*, and 1194 bp for *SmANS* showed that they share the presence of common motifs such as auxin, circadian rhythm, light, stress and phytohormone responsive elements, along with several MYB regulatory elements. Moreover, several sugar responsive elements were found in the promoters of *SmPAL*, *SmHQT*, and *SmANS*, whereas sugar starvation or hormone signaling motifs were not found in the *ANS* promoter (**Table 1**). Along with the structural genes, the isolated promoter region of *S. melongena Myb1* was compared with the promoter regions of other four MYBTF belonging to group 6 (Liu et al., 2015), namely *S. lycopersicum ANT1*, *S. tuberosum AN1* and *CAI*, and *V. vinifera MybA1*, whose sequences were retrieved from the respective genomic resources. Interestingly, the comparison between the TFs revealed that only MYBST1, MYBGAH, and MYBAT consensus were present in *SmMyb1*, while all the other putative MYB binding sites corresponding to MYBPLANT (MACCWAMC), MYBPZM (CCWACC), MYCATERD (CATGTGG), and MYBCORE (CNGTTR) were absent (**Table 1**). Moreover, some distinctive elements, such as elements for cell proliferation and growth, were found only in *SmMyb1* and *VvMybA1* promoters, while phosphate starvation responsive elements were present only in *S. tuberosum* and *S. melongena* TFs. Unique elements were found in the *SmMyb1* promoter, such as the TATCCAT motif, which is required for alpha-amylase expression during sugar starvation, as well as the SURE motif, shared only with *SmPAL* and *SmHQT* structural genes.

**Table 1:** List of common *cis-*acting regulatory elements for structural biosynthetic genes, *SmMYB1* and TF from other species.

IUPAC Family		*SmANS*	*SmHQT*	*SmPAL*	*SlANT1*	*StAN1*	*StCai*	*VvMYBA1*	*SmMYB1*	Function
PFAM014		1	3	7	3	3	3	3	3	MybSt1
PFAM171		3	0	3	0	4	0	2	0	Myc
P$FAM003		3	4	4	3	2	3	2	0	MybPLANT
Pfam170		4	0	0	0	0	4	4	0	MybPZM
Pfam266		1	3	1	2	2	5	4	3	MybAT
Pfam325		0	0	2	0	3	1	0	0	MYBCOREATCYCB1
		3	1	1	1	0	0	1	2	Mybgah
PFAM099		0	0	0	0	0	0	0	2	Phytocrome regulation
PFAM08		2	3	4	0	8	1	1	3	Plastid regulation
PFAM302		0	0	0	0	0	0	1	3	Cell proliferation and growth
PFAM234		0	0	0	2	0	1	0	1	Sporamine
										**Defense signaling and Wounding**
PFAM002		6	1	4	6	17	3	16	4	Wounding stress jasmonateinduction
PFAM010		5	3	5	6	2	3	5	4	WRKY and SalicylicAcid
PFAM322		5	2	3	3	0	1	6	2	Disease
PFAM290		7	8	8	11	0	5	2	12	Pathogen and Salt induced
										**Plant stress signaling**
PFAM292		1	1	1	0	0	1	1	1	Hypo osmolarity-responsive element
PFAM310		1	3	2	1	0	0	0	3	Cytokinin
PFAM266-026		5	4	1	4	2	7	9	3	Abscisic acid and Aba mediation
PFAM260-170		4	1	2	1	0	0	3	2	Gibberellin responsive
PFAM204		1	1	1	0	0	1	0	1	Gibberellin and abscisic acid
PFAM205		1	4	3	4	2	5	3	3	Gibberellin and sugar repression
PFAM107-273-025		0	1	2	0	0	0	1	3	Sugar starvation and hormone regulation
PFAM272		1	1	0	0	0	0	0	1	Binding amylase
PFAM267-098		16	10	6	2	3	6	5	7	Auxin/Auxine response
PFAM295		0	0	0	0	4	4	0	2	Phosphate starvation response
PFAM311		4	0	3	2	0	1	4	5	Low Co_2_
PFAM124		2	1	2	0	0	0	1	2	Ethylene responsive elements
PFAM305		1	0	1	0	0	2	0	2	Fermentative pathway
										**Light responsive *cis*-acting elements**
PFAM012-027		6	17	12	3	4	6	1	5	Light responsiveness /light regulation
PFAM262		2	3	2	2	7	2	1	1	Circadian expression/light
PFAM300		0	2	0	0	1	1	1	4	Sorlip

### Transient Expression in *N. benthamiana*

**FIGURE 5:**
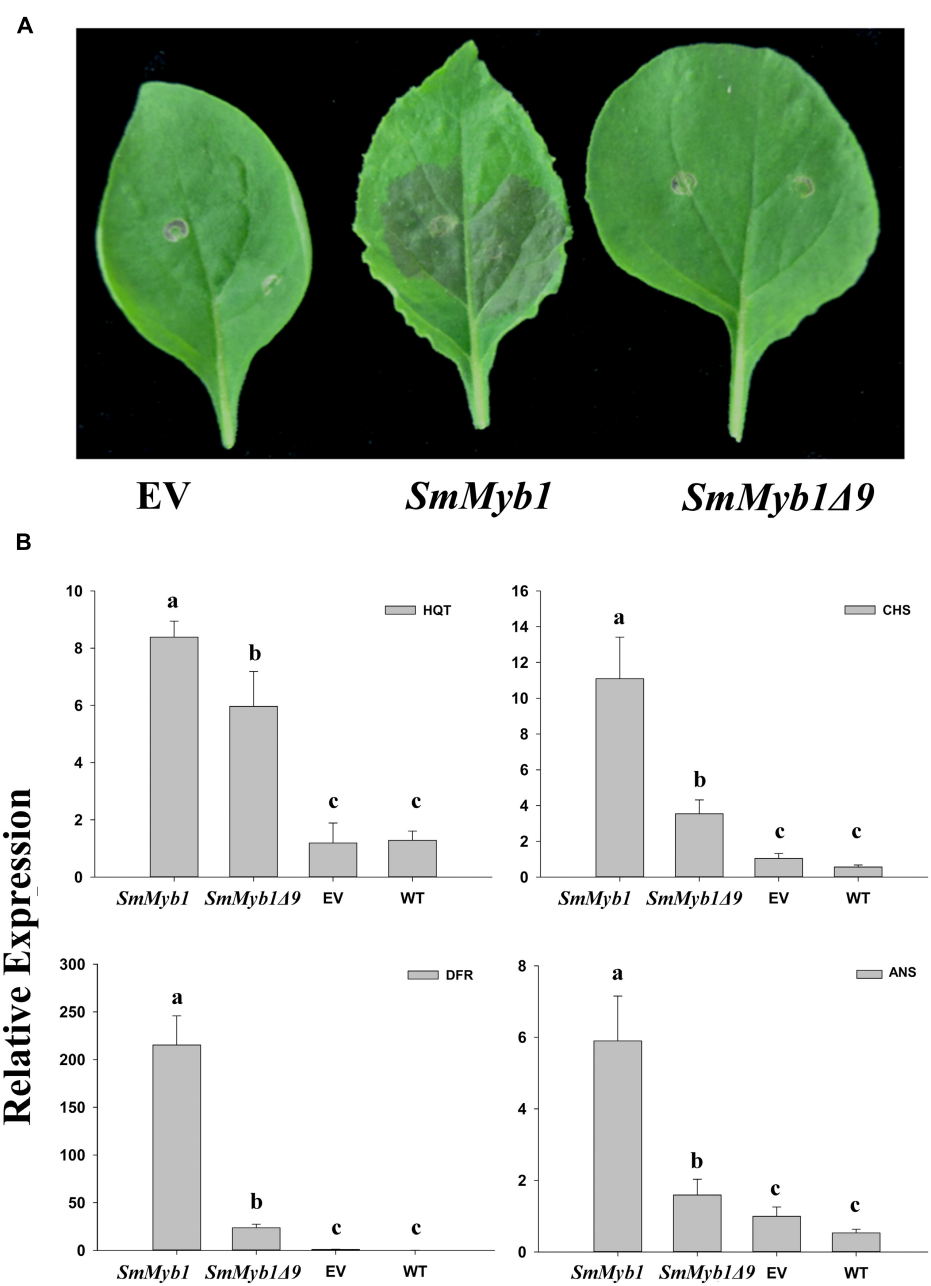
**The effects of over-expression of *SmMyb1* in *Nicotiana benthamiana* leaves. (A)** Leaves of *N. benthamiana* after agro-infiltration with *SmMyb1*, *SmMyb1Δ9*, pGWB411 (empty vector, EV). **(B)** Gene expression analysis of *HQT, CHS, DFR*, and *ANS* late phenylpropanoid structural genes in agro-infiltrated *N. benthamiana* leaves monitored by qRT-PCR. The results were analyzed using the ^ΔΔ^Ct method and presented as fold changes compared with the young leaves, used as internal calibrator. Data are reported as means ± SD. Means denoted by the same letter did not differ significantly at *p* ≤ 0.05 according to Duncan’s multiple range test.

The role of the isolated *SmMyb1* in the regulation of the phenylpropanoid biosynthetic pathway was investigated through transient transformation of *N. benthamiana* leaves of the full length *SmMyb1* gene. *SmMYB1Δ9*, a truncated mutant obtained by deleting nine C-terminal triplets from the *SmMyb1* sequence, was also transformed in *N. benthamiana* leaves to study the functional role of the conserved C-terminal domain. Both the full length and the truncated genes were cloned into the transient expression Gateway vector pGWB411, transfected into *Agrobacterium* and infiltrated into *N. benthamiana* leaves, alongside with the empty vector. Five days post inoculation, *SmMyb1* agro-infiltrated *N. benthamiana* leaves showed an anthocyanic-pigmented phenotype, which was clearly visible due to the lack of anthocyanic pigmentation in wild type tobacco leaves. On the contrary, *SmMyb1*Δ9 as well as the empty vector-infiltrated leaves did not show any red pigmentation (**Figure 5A**). Beside visible anthocyanin accumulation, metabolic analysis showed that *SmMyb1* over-expression induces a strong accumulation of D3R (130.21 ± 20.56 μg/100 mg dw), which was barely detectable in *SmMYB1Δ9* agro-infiltrated leaves (14.95 ± 3.04 μg/100 mg dw) and not detectable in the empty vector agro-infiltrated controls. Interestingly, a CGA content of 835.09 ± 60.06 and 792.00 ± 50.03 μg/100 mg dw was found in *SmMyb1* and *SmMyb1*Δ9 agro-infiltrated leaves, respectively, an almost doubled amount in comparison to what was found in the empty vector transformed and in untransformed leaves (464.80 ± 43.71 μg/100 mg dw, 354.67 ± 13.34 μg/100 mg dw, respectively, **Table 2**).

**Table 2:** Metabolite content in *Nicotiana benthamiana* agro-infiltered leaves with *SmMyb1*, *SmMyb1Δ9*, EV (Empty Vector), and WT (Wild Type).

*N. benthamiana leaves*	D3R (μg/100 mg Dw)	CGA (μg/100 mg Dw)
*SmMyb1*	130.21 ± 20.56	835.09 ± 60.06^a^
*Sm Myb1Δ9*	14.95 ± 3.04	792.00 ± 50.03^a^
EV	Nd^∗^	464.80 ± 43.71^b^
WT	Nd^∗^	354.67 ± 13.34^c^

*^∗^Nd, not detected compounds.*

*Data are reported as means ± SD. Means denoted by the same letter did not differ significantly at p ≤ 0.05 according to Duncan’s multiple range test.*

Expression analysis of several key phenylpropanoid biosynthetic genes, i.e. *HQT, CHS, DFR*, and *ANS*, detected a similar expression level of the *SmHQT* gene in *SmMyb1* and *SmMyb1Δ9* agro-infiltrated leaves, whereas higher levels of expression for *CHS*, *DFR*, and *ANS* were measured in *SmMyb1* leaves in comparison with *SmMyb1Δ9* transformed leaves (**Figure 5B**). In agreement with previous studies (Spelt et al., 2000; Kiferle et al., 2015), a strong induction of the *DFR* gene was detected in *SmMyb1* agro-infiltrated leaves, about 10 and 100 times higher than in *SmMyb1Δ9* agro-infiltrated and control leaves.

### Protein–Protein Interaction

**FIGURE 6:**
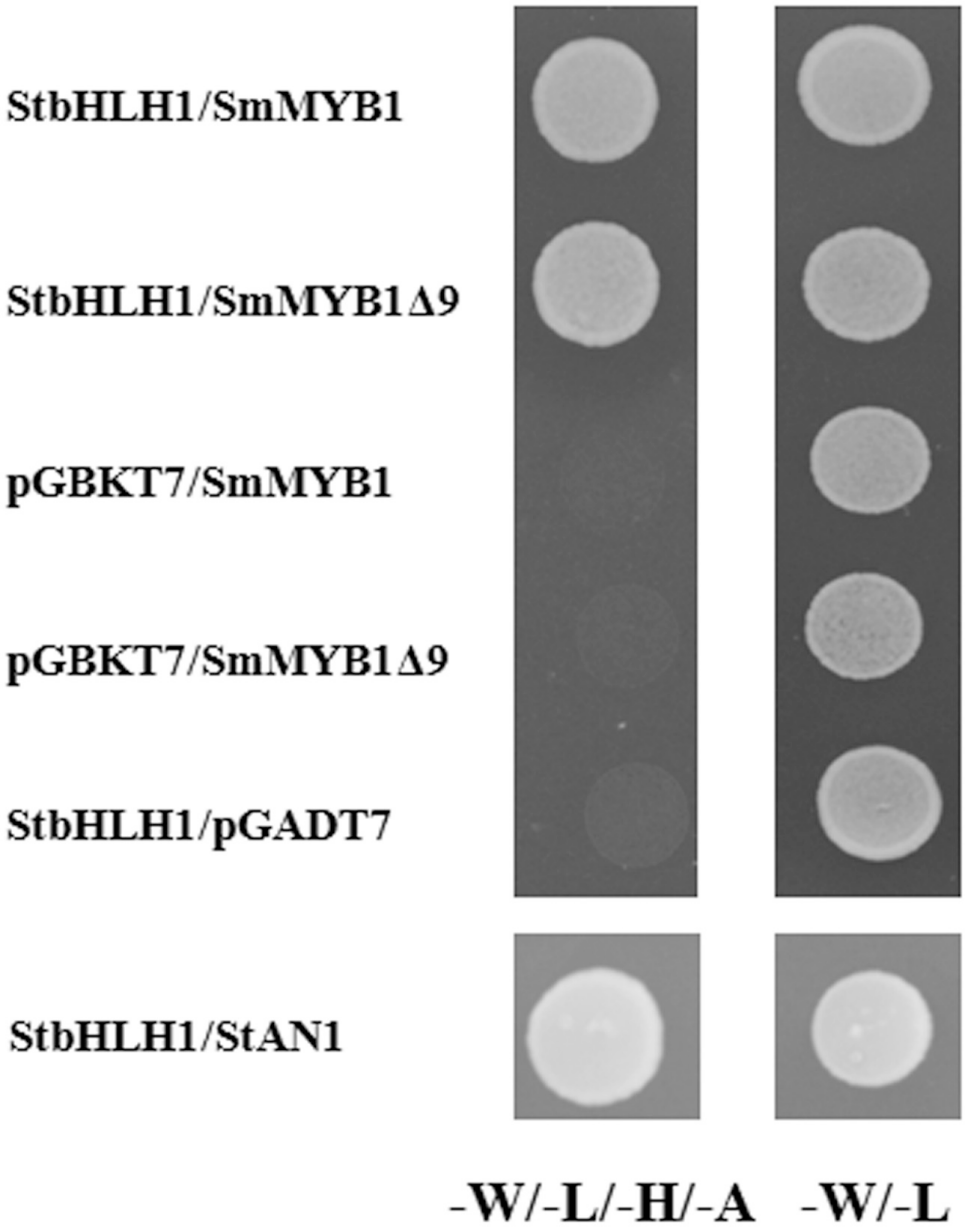
***Sm*MYB1 interacts with bHLH TF *Stb*HLH1 in the yeast two-hybrid assay.** *SmMYB1* and *SmMyb1*Δ9 were cloned in the prey plasmid pGADT7 and co-transformed with StbHLH1pGBKT7 (D’Amelia et al., 2014) in yeast strain AH109. The *SmMyb1*pGADT7/pGBKT7 and pGADT7/*StbHLH1*pGBKT7 combinations served as negative controls while *StbHLH1*/*StAN1* is shown as positive control. Yeast cells grown on synthetic complete media (–W/–L, **Right**) and on selective media (–W/–L/–H/–A, **Left**) are shown. Pictures were taken 3 days after incubation at 30°C.

The regulatory function of several MYB proteins in anthocyanin biosynthesis depends on their ability to form a regulatory complex with bHLH partners (Lin-Wang et al., 2010; Pattanaik et al., 2010; Albert et al., 2014). To determine the ability of *SmMYB1* to interact with heterologous bHLH proteins, we performed a yeast two-hybrid assay to verify interaction with a previously identified *StbHLH1* from potato. This particular bHLH was selected because its role in anthocyanin regulation is well established both in tubers and leaves of potato (Payyavula et al., 2013; D’Amelia et al., 2014). Because a previous report had shown that fusion of an anthocyanin MYB-type regulator from petunia with the GAL4 binding domain (GAL4 BD) resulted in auto activation of the reporter genes *HIS* and *ADE*, while fusion with the GAL4 activation domain (GAL4 AD) did not (Quattrocchio et al., 2006), we fused *SmMYB1* or a truncated form lacking the last nine amino acids (*SmMyb1Δ9*) with GAL4 AD. After preliminary assessment of the absence of auto-activation of reporter genes for all the used constructs (Supplementary Figure S5), the interaction of *SmMYB1* or *SmMYB1Δ9* with *StbHLH1* fused with GAL4 BD was verified. As shown in **Figure 6**, yeast cells co-transformed with *SmMyb1* and *StbHLH1* were capable of growing on selective media lacking leucine, tryptophan, histidine, and adenine. Negative controls, consisting of yeast cells co-transformed with prey plasmids containing *SmMyb1* and empty bait plasmid, as well as the opposite combination, *StbHLH1* combined with empty prey plasmid, did not grow on selective medium, indicating that an interaction between *SmMYB1* and *StbHLH1* does take place in yeast. *SmMYB1*Δ9 was still capable of interaction with *StbHLH1*, suggesting that the tested C-term truncation did not interfere with the interaction, as expected by previous reports showing that interaction with bHLH partners requires the N-terminal portion of MYB-type TFs (Plazas et al., 2013a).

## Discussion

*Solanum melongena* is placed in the top rank among the edible Solanaceae and other vegetables with high radical scavenging properties. These beneficial traits are due to the high accumulation of antioxidant polyphenols in eggplant fruit flesh and skin (Plazas et al., 2013a). The most important phytonutrients in this species are CGA, and the anthocyanic pigments, delphinidin 3- rutinoside and/or nasunin (Mennella et al., 2010). Besides the benefits to the human health (Jaganath and Crozier, 2009), these compounds play an active role in the plant defense against biotic and abiotic injuries (Hura et al., 2008) and thus their synthesis must be tightly regulated.

The importance of these specialized metabolites prompted us to investigate phenylpropanoid biosynthesis and its regulation in eggplant. In this study we report our findings on metabolite distribution, transcripts accumulation, along with a characterization of biosynthetic genes and a special focus on a R2R-MYB putatively involved in transcriptional regulation of biosynthetic genes.

### Phenylpropanoid Accumulation and Expression Analysis of Structural Genes

Metabolic analysis showed CGA accumulation in all the analyzed tissues of the Occidental eggplant cv. “Lunga Napoletana.” The highest content of CGA, of about 4000 μg/100 mg dw, was found in fruit flesh and skin, while almost half the amount was detected in all the other tissues. However, it is roughly 10 and 100 times higher than in tomato and potato, respectively. Although a wide variation of CGA content has been reported in the eggplant gene pool (Plazas et al., 2013b), our data confirm that this vegetable is the best source of CGA among Solanaceous species (Plazas et al., 2013a).

The flavonol rutin was barely detectable in leaves at two developmental stages and in stems, and slightly higher in the fruit skin, where D3R was found in high amounts, similarly to flowers, another pigmented tissue. The D3R content detected in the fruit skin of our eggplant cultivar is consistent with the amounts reported by Mennella et al. (2012) for non-Japanese genotypes.

Overall, our metabolic analysis indicates the eggplant fruit, and in particular the fruit skin, as the major accumulator of nutraceutical compounds.

Transcription profiles of flavonoid and CGA structural genes supported metabolic analyses. Early genes of the phenylpropanoid pathway were found expressed in all tissues, with higher transcript levels detected in fruits. However, *SmPAL* and the late gene *SmHQT* showed an order of magnitude higher expression levels in the fruit skin than *C4H* and *4CL*, which mirrored the higher accumulation of CGA in this tissue. This result may reflect the different functions of these genes. PAL, C4H, and 4CL enzymes, as initial committed steps in phenylpropanoid formation, can play multiple functions by providing phenylalanine-derived units for the different branches of the pathway, while HQT is specifically responsible for CGA biosynthesis. However, the coordinated expression (correlation coefficient *r* = 0.756, *p* < 0.05) of the eggplant *PAL* and *HQT* genes isolated in this paper may account for the high accumulation of CGA in eggplant, similarly to what was demonstrated for specific *HQT* and *PAL* isoforms in tobacco and tomato (Niggeweg et al., 2004; Payyavula et al., 2014). In pigmented tissues, like fruit skin and flowers, extremely high expression levels were detected for the flavonoid structural genes, *DFR* and *ANS*, which correlated with the D3R content (*r* = 0.991, *p* < 0.05, and *r* = 0.992 *p* < 0.05, respectively). As shown in tomato, the fruit surface accumulates a vast array of secondary metabolites, which are necessary for the fruit survival (Mintz-Oron et al., 2008), but whether metabolite accumulation in the fruit peel is the result of *de novo* biosynthesis or of active transport remains unclear. The correlation between accumulation of key structural gene transcripts and of the corresponding metabolites in the eggplant fruit skin suggest that this tissue might have an active role in their biosynthesis, although more accurate studies, e.g., isotope labeling (Docimo et al., 2012) or epidermis cell enrichment by laser dissection technologies combined with transcriptomic and metabolic profiling, would be necessary to definitively clarify this point.

### Isolation of CGA Biosynthetic Genes and of a MYB Regulatory Gene

To gain knowledge on the accumulation of CGA, we firstly searched for the candidate biosynthetic genes, whose sequences were not publically available from the eggplant draft genome at the time of these experiments. Besides, draft genomes are known to be less complete than finished genomes, and to be prone to misassembling and sequencing errors. Therefore, the full length cDNA sequences of *SmPAL* and *SmHQT* were isolated by conventional 3′ 5′ RACE from eggplant fruit flesh tissue. The PAL protein is encoded by a multi gene family, which encountered extensive duplications during evolution. About 18 and 13 *PAL* sequences are found in the potato and tomato genomes, respectively (Albert and Chang, 2014). Blast analysis in the draft genome indicated that several *PAL* partial sequences were present, which, however, showed a relatively low similarity level in respect to homologs sequences from “sister species,” confirming the extensive genetic variation already reported in eggplant varieties (Li et al., 2010). According to our phylogenetic analysis (Supplementary Figure S1) performed on homologous sequences from other plant species including Solanaceae, *Sm*PAL is closely related to the *C. annuum*, *S. tuberosum*, and *S. lycopersicum* ones, and its structure mostly resembles a PAL1-like protein (Joos and Hahlbrock, 1992). Similarly, *Sm*HQT resulted phylogenetically grouped with *S. tuberosum* HQT, *S. lycopersicum* HQT, and *N. tabacum* HQT (Supplementary Figure S2). Although from the sequence similarity it is not possible to predict whether shikimate or quinate might be the preferential substrate for this enzyme, shikimate esters were not detectable in our analyses, thus indicating this eggplant HQT as a true HQT (Comino et al., 2009; Sonnante et al., 2010; Lallemand et al., 2012; Pardo Torre et al., 2013). Unlike potato tubers (Payyavula et al., 2012), *SmHQT* expression in eggplant correlates with CGA accumulation, suggesting that the major route for CGA formation in eggplant might be through HQT, as reported in tomato and tobacco (Niggeweg et al., 2004). Deeper comparative analysis would be necessary to evaluate the level of conservation among the sequences isolated in this work in respect to those of the eggplant draft genome. Nevertheless, it is worth noting that the presence of several SNPs might underlie that eggplant lines geographically unrelated (Asian cvs. versus Occidental cvs.) have encountered a different evolutionary program.

Along with CGA, also the high anthocyanin content contributes to the sensorial and nutraceutical properties of eggplant fruits, as well as to improved plant tolerance to biotic and abiotic stresses. Correlation between fruit color and improved quality has been reported for many species, and it is known that higher anthocyanins content in tomato fruits reduces pathogen susceptibility (Bassolino et al., 2013; Zhang et al., 2013). Therefore, knowledge of the factors controlling the production and distribution of CGA and anthocyanins is of great moment for genetic improvement of plant species.

To provide insights into the regulation of phenylpropanoid production in eggplant, we searched for a MYB TF homologous to *S. tuberosum* CAI, which was shown to be a regulator of CGA and flavonoids biosynthesis (Rommens et al., 2008). We isolated a MYBTF from the *S. melongena* cv. ‘Lunga Napoletana,’ which resulted to contain four SNPs in respect to *Sm*MYB1 from the cv. ‘Zi Chang’ (Zhang et al., 2014), determining two non-synonymous amino acid transitions, possibly affecting the function of the encoded protein. Interestingly, also the draft eggplant genome contains a nucleotide sequence with four SNPs, encoding a protein identical to our sequence, which was annotated as ANT1 (Hirakawa et al., 2014).

Similarly to several R2R3-MYB proteins-encoding genes, such as *PhAN2*, *NtAN2*, and *PAP1*, also *SmMyb1* shares a conserved intron/exons organization, thus supporting the idea that they might have a common evolutionary origin (Quattrocchio et al., 1999; Borevitz et al., 2000; Pattanaik et al., 2010).

The alignment with 12 highly similar R2R3-MYB TF showed that *Sm*MYB1 shares all the typical features of a MYB anthocyanin biosynthesis activator (Supplementary Figure S3), a bHLH interaction domain, a ANDV domain, as well as the conserved sequence KPRPRS/TF at the end of the R3 domain (Stracke et al., 2014). As most MYB proteins of this class, *Sm*MYB1 retains the residues FXXXDLVS at the C-terminal, whose function, contrarily to MYB repressor proteins (Dubos et al., 2008, 2010; Matsui et al., 2008; Albert et al., 2014; Xu et al., 2014) has been investigated to a lesser extent.

Further, we investigated the phylogenetic relationships of *Sm*MYB1 with 28 related R3R2-MYB proteins involved in the activation of phenylpropanoids. Neighbor Joining analysis placed *Sm*MYB1 in a clade with other sequences from Solanaceae, namely *S. tuberosum* Chlorogenate inducer CAI, *S. tuberosum* AN1 and *S. lycopersicum* ANT1, suggesting that *SmMyb1* is an eggplant homologous of *SlANT1*. Moreover, a BLAST analysis of *SmMyb2* (Zhang et al., 2014) onto the tomato genome indicated that this gene is located, together with the Heat Shock-encoding gene *SmHsp70-2-like* and with several candidate genes for anthocyanin accumulation, on chromosome 10, in a QTL controlling anthocyanin pigmentation (Doganlar et al., 2002; Barchi et al., 2012; Fukuoka et al., 2012), and is syntenic with *SlAN2* (Tomato Genome Consortium, 2012). Consistently, *Sm*MYB2 clusters closely to AN2 from *S. lycopersicum* and *S. tuberosum*, thus suggesting a possible distinct regulatory role from *Sm*MYB1. Eggplant R2R3-MYB TFs homologous to *SlANT1* and *SlAN2* were recognized as main regulators of anthocyanin pigmentation (Kiferle et al., 2015). Nevertheless, the *SlANT1*-homologous potato gene *StAN1* was also shown to have a key role in phenylpropanoid accumulation, namely in regulating CGA synthesis in potato (Payyavula et al., 2014). To determine the involvement of *Sm*MYB1 in the regulation of phenylpropanoid accumulation, we measured the expression levels of *SmMyb1*, *SmMyb2*, *SmTT8* and *SmHsp70-2-like*. Except for stems, *SmMyb1* expression was overall low in all tissues, while *SmMyb2* transcripts accumulated at high levels in anthocyanic tissues, and especially in flowers, confirming previous data on *SlANT1* and *SlAN2*, respectively, in tomato (Kiferle et al., 2015). The bHLH-encoding *TT8* and *HSC70-2-like* genes resulted to be highly expressed in stems and flowers, and even more in the fruit flesh and skin, where their expression levels were about 10 to 100 times higher than the analyzed MYBs. These results strongly supported the involvement of these two genes in anthocyanin accumulation.

Anthocyanins are known to contribute to stress resistance in plants (Chalker-Scott, 1999; Lev-Yadun and Gould, 2009). During heat stress, anthocyanins are produced to decrease leaf osmotic potential and prevent loss of water, while bHLH proteins participate to heat-related mechanisms and hormone signaling (Leivar and Quail, 2011) and heat shock proteins function in avoiding protein misfolding (Bita and Gerats, 2013). It is tempting to speculate that TT8, HSC70-2 like and anthocyanins take part to protective mechanisms toward the gradual increase in temperature experienced by ripening fruits. However, elucidation of the functions that TT8, HSC70-2 like and anthocyanins may play during eggplant development or stress response requires further investigation. Our biochemical and expression data, together with the sequence homology between *SmMyb1* and *SlANT1* suggest that the TF gene isolated in this study is somehow involved in the control of anthocyanin pigmentation by taking part in the MBW complex, although with a more marginal role than hypothesized by Zhang et al. (2014), and in accordance to the recent reports on *SlANT1* in tomato (Kiferle et al., 2015).

Since CGA and anthocyanin production is modulated by biotic and abiotic factors, we searched the promoter regions of *SmPAL*, *SmHQT*, *SmANS*, and *SmMyb1* for relevant *cis*-acting elements. Multiple *cis*-acting elements, including fundamental and special elements associated with defense signaling and hormone regulation were found in the *SmMyb1*, *PAL*, *HQT*, and *ANS* promoters. The presence of the same light, circadian rhythm and sucrose responsive elements in *S. melongena* phenylpropanoid genes and *SmMyb1* promoters suggests they may be coordinately expressed and supports the idea that in eggplant CGA and anthocyanins accumulation is controlled by the same environmental factors as in potato tubers (Payyavula et al., 2013). This is consistent with the CGA and anthocyanin role in plant biotic interactions (Dixon, 2001; Del Campo et al., 2013), and their abundance in eggplant tissues suggests that this specialized metabolites might actively participate in inducible defenses, either by triggering plant resistance to pathogens or functioning as donor of structural elements for cell wall formation in case of damage (Malinovsky et al., 2014). A BLAST analysis of the ‘Lunga Napoletana’ promoter sequences in the draft genome of the Asian cv. ‘Nakate-Shinkuro’ highlighted that, beside the ANS promoter, all the other 5′ upstream regions have a lower level of similarity (data not shown), thus suggesting that general phenylpropanoid gene regulation may be influenced by distinct regulatory signals in the two eggplant varieties.

Extension of the comparative analysis to the promoters of other anthocyanins and phenolic acids-related TFs, *SlANT1*, *StAN1*, *StCAI*, and *VvMybA1*, detected distinctive regulatory motifs in the *SmMyb1* promoter. Several elements for ethylene, cytokinin and gibberellin responsiveness were found, which were scarcely represented or absent in *SlANT1* and in the potato *AN1* and *CAI* TFs promoters, thus suggesting that this eggplant TF might sense hormone signaling and mediate phenylpropanoids production as an active response to abiotic and biotic stresses (Croteau et al., 2000; Gális et al., 2006). Moreover, the presence of additional and distinctive elements involved in the response to phosphate/sugar starvation, phytochrome/plastid regulation, sporamine formation and cell proliferation and growth gives an indication that the activation of this TF is induced by different factors than the other TFs and that it may play various and different physiological roles in eggplant.

The functional role of *SmMyb1* in phenylpropanoid biosynthesis regulation was further tested by transient overexpression in *N. benthamiana* leaves. It is known that sequence variability at the conserved C-terminal region of *PhAN2*-*like*, as well as *C1* from maize, is tolerated without affecting protein functionality, while mutation or nucleotide variation determining premature stop codon results in a complete loss of activity (Goff et al., 1992; Quattrocchio et al., 1999). However, the function of this domain has not been elucidated so far. To address this point, we performed functional analysis of a *SmMyb1* C-terminal truncated form. Opposite to *SmMyb1Δ9* and empty vector-transformed leaves, *SmMyb1* over-expression determined a red pigmentation of tobacco leaves, which normally accumulate very low amounts of anthocyanins. The red leaf phenotype correlated both with a high expression level of the late anthocyanin biosynthetic gene *DFR* and with a higher content of the D3R pigment. Additionally, the normal phenotype of the tobacco leaves carrying the MYBTF truncated form was consistent with the lower expression of the *DFR* gene and with a barely detectable D3R content. These results confirmed that anthocyanin regulation by *SmMyb1* proceeds thorough the activation of *DFR* transcription, as it was shown for *SlANT1* and *SlAN2* (Kiferle et al., 2015). Interestingly, *SmMyb1* and *SmMyb1Δ9* transformed leaves also showed higher expression of *CHS*, *HQT*, and *ANS*, along with a doubled content of CGA. These results suggest that, similarly to *StAN1*, *SmMyb1* may have a direct involvement in CGA biosynthesis and that a deletion at the C-terminal determines a loss of activity on anthocyanin biosynthesis. Therefore, we may speculate that the C-terminal domain in *Sm*MYB1 is essential for transcriptional activation of anthocyanin genes, though its regulative function on the production of other metabolites, like CGA, is not compromised by the mutation.

Remarkably, we found a high accumulation of *HSC70-2* like and *TT8* in both anthocyanic and non-anthocyanic eggplant tissues, thus suggesting that these anthocyanin-related genes (Barchi et al., 2011) are not the limiting factor for anthocyanin accumulation, but require parallel MYBs expression to promote their synthesis via the MBW complex in *S. melongena* flower and fruit skin.

Two-hybrid interaction indicated that the *SmMyb1* and its truncated form are both able to interact with a heterologous bHLH, but results of the transient overexpression of *SmMyb1* without bHLH suggests that the eggplant TF is able to recruit a tobacco endogenous partner (Quattrocchio et al., 1998; Pattanaik et al., 2010). Moreover, these results indicate that the *SmMyb1* TF alone is sufficient to trigger anthocyanin accumulation

## Conclusion

We have improved our knowledge on the behavior of phenylpropanoid genes in eggplant, and demonstrated the role of *SmMyb1* in controlling both anthocyanin and CGA synthesis in *S. melongena* tissues. Besides, for the first time, we propose a functional role of the C-terminal domain of this TF. Our results may thus contribute at facilitating and improving the design of targeted breeding strategies and metabolic engineering approaches to increase accumulation of specific antioxidant and color-related phenolics in target species.

## Author Contributions

TD and MT designed research; TD, AR, GB, and MDP performed research; GM and GF designed and performed biochemical analyses; TD, GB, and MT analyzed data; LB, LT, and GR provided bio-informatic analyses and critical suggestions; TD and MT wrote the paper.

## Conflict of Interest Statement

The authors declare that the research was conducted in the absence of any commercial or financial relationships that could be construed as a potential conflict of interest.

## Footnotes

1 http://eggplant.kazusa.or.jp/

2 http://www.primmbiotech.com

3 http://www.expasy.ch/tools/scanprosite/

4 http://www.genome.jp/tools/clustalw/

5 www.sigmaplot.com

6 https://www.genomatix.de

## Abbreviations

bHLH: basic helix-loop-helix
DNA: deoxyribonucleic acid
LC–MS: liquid chromatography–mass spectrometry
qRT-PCR: quantitative real time-polymerase chain reaction
RNA: ribonucleic acid
TF: transcription factor
