# Supplementary material for: Phenylpropanoids Accumulation in Eggplant Fruit: Characterization of Biosynthetic Genes and Regulation by a MYB Transcription Factor
Source: Front Plant Sci. 2016 Jan 28;6:1233. doi: 10.3389/fpls.2015.01233 (PMC4729908; doi:10.3389/fpls.2015.01233)
Supplement: Supplementary file 1 [file Data_Sheet_1.PDF]

Supplementary Material

Phenylpropanoids accumulation in eggplant fruit: characterization of biosynthetic genes and regulation by a MYB transcription factor.

Docimo T[1]*., Francese G[2]., Ruggiero A[1]., Batelli G[1]., De Palma M[1]., Bassolino L[3]., Toppino L[3]., Rotino G.L[3]., Mennella G[2]., Tucci M[1]*.

[1]Consiglio Nazionale delle Ricerche, Istituto di Bioscienze e BioRisorse, UOS Portici, Via Università 133, 80055 Portici, NA (Italy)

[2]Consiglio per la ricerca in agricoltura e l'analisi dell'economia agraria, Centro di Ricerca per l'Orticoltura (CREA-ORT), Via dei Cavalleggeri 25, 84098 Pontecagnano, SA (Italy)

[3]Consiglio per la ricerca in agricoltura e l'analisi dell'economia agraria, Unità di Ricerca per l'Orticoltura (CREA-ORL), Via Paullese 28, 26836 Montanaso Lombardo, LO (Italy)

Supplementary Table 1 List of primers used in this study

Gene	Accession number	Sequences	Orientation	Scope
SmC3H	FS013298.1	GAATGGACACAACTGCAATCTCT<br>GGAAGTCTGTTTCGTTCATCACA	For<br>Rev	qRT
SmPAL	FS058603.1	CGCACAGCAACTAAGATGATCG<br>GCAAGGGCCAATCTTGTATTATCC	For<br>Rev	qRT<br>3'5'RACE
GSP1PAL	FS058603.1	GACCCCACAACTTTTGTTATCAGCATTTGC	Rev	5'RACE
Sm4CL	FS021677.1	CCGGATACGGGTTGCTCTC<br>CCGGCGTGTAACCATCCT	For<br>Rev	qRT
SmHQT	FS083932.1	GTTGAGGCCCAAAGTGATTC<br>GACTCCGCCACAGCTAAAAC	For<br>Rev	qRT-<br>3'5'RACE
GSP2HQT	FS083932.1	TCATCCCTAGATAATCTCCCAGCCATTGG	Rev	5'RACE
SmC4H	FS082784.1	TGGCGATCCCTCTCTTAGTCC<br>CCAGTGAGCAGGGTTGTTGG	For<br>Rev	qRT
SmAPRT	FS056270.1	TGCATGTAGGTGCTGTGCAAG<br>ACGCTCAAGAAGCCTAATCGC	For<br>Rev	qRT
SmMyb1	FS084890	GGACCGCAAACGATGTAAAG<br>TTTCCGAGGTTGAGGTCTTATT	For<br>Rev	qRT
SmPAL	KT259041	ATGGAGTCAATTGCACAAAATGTACATG<br>CTAGCAGATTGGAAGAGGAGCACCAT	For<br>Rev	sequencing
SmHQT	KT259042	ATGAAAATTAGTATCAAAGAATCAACACTAG<br>AAGGTCATACAAGTACTTTTCGAATAGTGGC	For<br>Rev	sequencing
SmANS	EU809469.1	GATTGGGTTGGGATTGGA<br>TAGTTCTGGTTGGGGGCATT	For<br>Rev	qRT
SmDFR	FS074352.1	AGGACCCTGAGAATGGAGTAA<br>TCAAGAGTTCCAGCAGATGAAG	For<br>Rev	qRT
SmHSC70-2like	KT591487	GCCATTGAGCAAGCCATT<br>CATCCATGGCACCACCGT	For<br>Rev	qRT
SmTT8	KT591486	TTGCCCAGACGGTAGAAC<br>CAGCCGACCCAACCCCACTT	For<br>Rev	qRT
SmMyb1	FS084890	TGGTGAAGGCAAGTGGCATCTT<br>GTGACTTTGCTTCGGATGAAGTGG	For<br>Rev	qRT<br>3'5'RACE
SmMyb1	KT259043	ATGAATAATCCTCCTATAATCTGTACGTCTG<br>TTAATCAAGTAAATTCCATAAATCAATATCA	For<br>Rev	sequencing
pGWB411:Myb1	KT259043	CACCATGAATAATCCTCCTATAATCTGTACGT<br>C ATCAAGTAAATTCCATAAATCAATATCA	For<br>Rev	Gateway cloning

ANSGSP1	EU809469.1	CCTTGTCCTCCGAGTCAATTTCTTTCAGA	Rev	Genome walking
ANSGSP2	EU809469.1	GCCAAGCTTTCAACTCTTGAAGGAGTTGG	Rev	Genome walking
MYBGSP1	KT259043	CAGACGTACAGATTATAGGAGGATTATTCAT	Rev	Genome walking
MYBGSP2	KT259043	CATGGCTATTTATATTTTCTGATGTTGATATC	Rev	Genome walking
StbHLH	HG763863	ATTACCCGGGTATGGAGATTATACAGCC ATTTAGTCGACTTAATTAGCTCTAGGG	For rev	Two Hybrid
EcoSmMyb1	KT259043	CAGTGAATTCATGAATAATCCTCCTATAATCT GTACGTCTG	For	Two Hybrid
XhoSmMyb1	KT259043	CGAGCTCGAGTTAATCAAGTAGATTCCATAA ATCAATATCA	Rev	Two Hybrid
XhoSmMyb1Δ9	KT259043	CGAGCTCGAGTCAGCAAAAAAAATCATCCCA ATTATCA	Rev	Two Hybrid

(A)

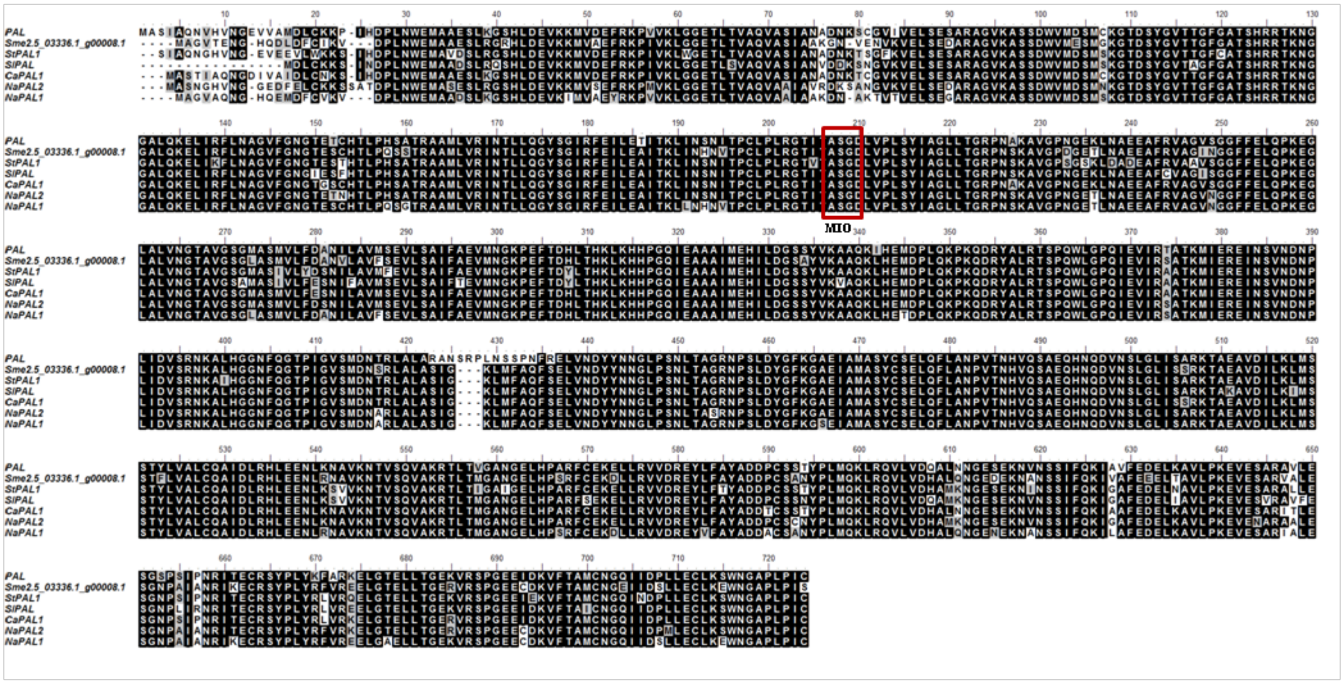

(B)

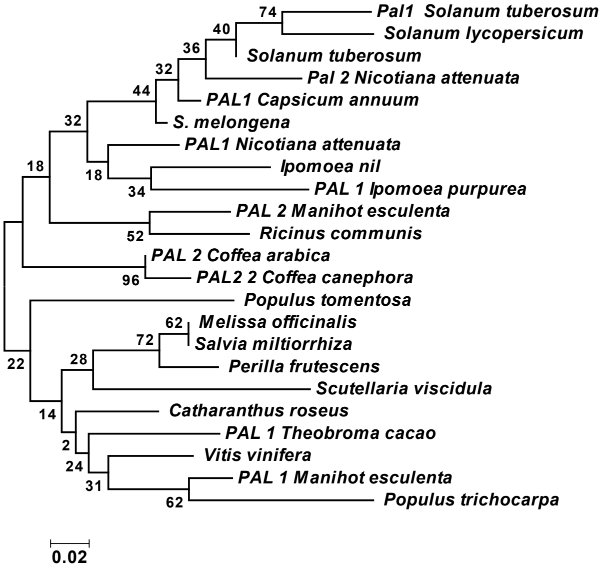

Supplementary Figure 1 (A) The amino acid sequence of *S. melongena* PAL was aligned with a highly similar PAL protein from the *S. melongena* draft genome (Sme2.5_03336.1_g00008.1) and with representatives of other Solanaceae, *S. tuberosum, S. lycopersicum, N. attenuata*, and *C. annuum.* The invariable motif Ala-Ser-Gly, known as MIO domain, is delimited with a solid line box within the conserved active site GTITASGDLVPLSYIA**. (B)** The PAL proteins identified from other species were aligned using Clustal X, and the PAL phylogeny was constructed using the neighbor-

joining method with the MEGA6 program. The branch lengths are indicated above the branch lines. Protein sequences used for phylogenetic analyses have the following accession numbers: *Capsicum annuum* CaPAL1 (AIA66448.1); *Solanum tuberosum* StPAL1 (P31425.1); *Solanum tuberosum* StPAL (AGT63063.1); *Nicotiana tabacum* NtPAL2 (ABG75911.1); *Solanum lycopersicum* SlPAL (AAA34179.2); *Nicotiana attenuata* NaPAL (ABG75910.1); *Ipomoea nil* InPAL (AAG49585.1); *Ipomoea purpurea* IpPAL1 (AHJ60264.1); *Catharanthus roseus* CrPAL (BAA95629.1); *Cynara cardunculus* CcPAL2 (AEO92028.1); *Ricinus communis* RcPAL (AGY49231.1); *Capsicum annuum* CaPAL2 (AEL21617.1); *Melissa officinalis* MoPAL (CBJ23826.1); *Perilla frutescens* PfPAL (AEZ67457.1); *Scutellaria viscidula* SvPAL (ACR56688.1); *Manihot esculenta* MePAL2 (AAK60275.1); *Salvia miltiorrhiza* SmPAL (ABD73282.1); *Manihot esculenta* MePAL1 (AAK62030.1); *Theobroma cacao* TcPAL1 (XP_007027354.1); *Vitis vinifera* VvPAL (AEX32784.1); *Populus trichocarpa* PtPAL (XP_002312013.1).

(A)

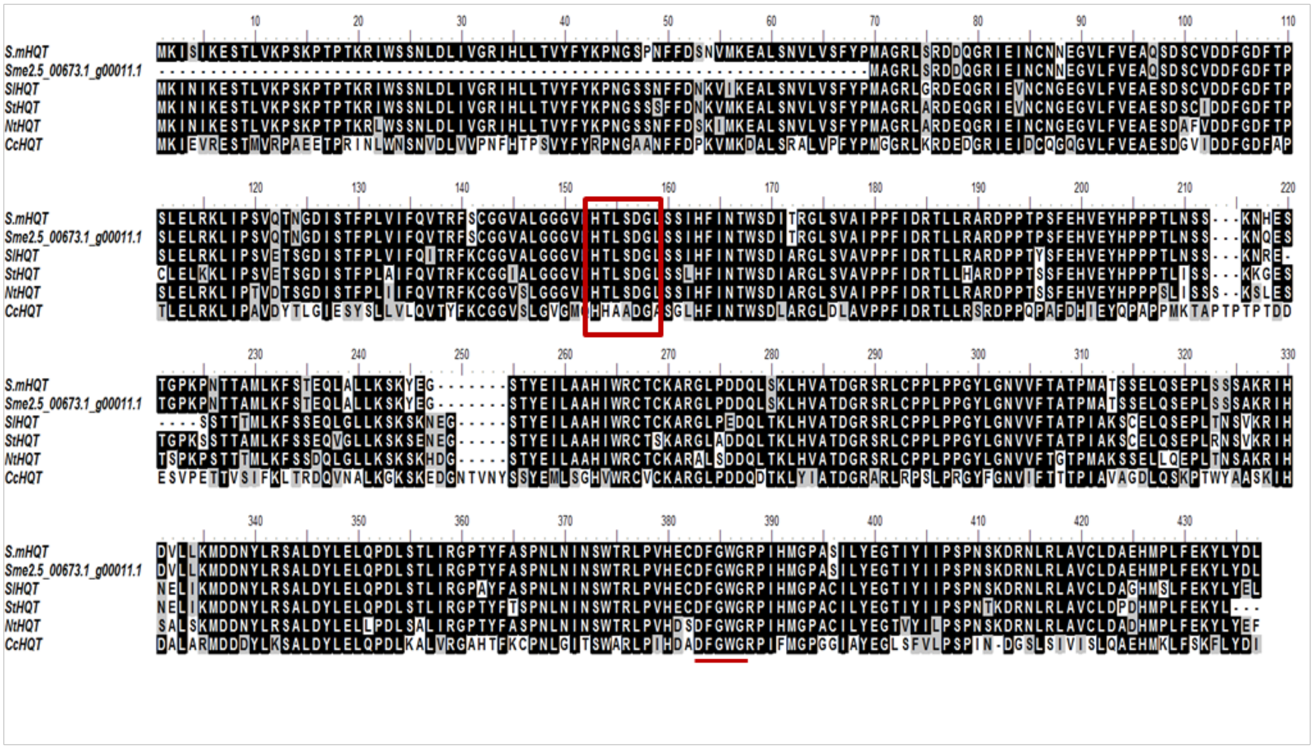

(B)

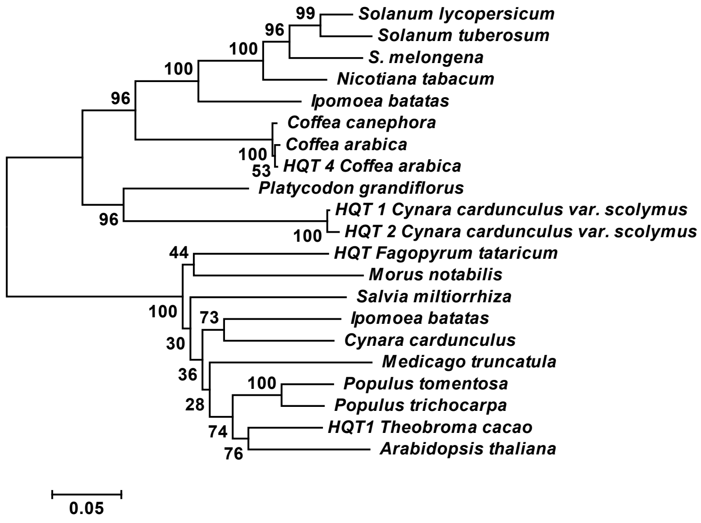

Supplementary Figure 2 (A) The amino acid sequence of *S. melongena* HQT was aligned with HQT found in the *S. melongena* draft genome (Sme2.5_00673.1_g00011.1), with representatives of other Solanaceae, *S. tuberosum, S. lycopersicum, N. tabacum*, and with *C. cardunculus* from Asteraceae. The conserved active site HXXXDG, characteristic of acyltransferase protein, is delimited by a red box, and the DFGWG block from position 383 to 389 is underlined. **(B)** The HQT proteins identified from other species were aligned using Clustal X, and the HQT phylogeny was constructed using the neighbor-joining method with the MEGA6 program. The branch lengths are indicated above the

branch lines. Protein sequences used for phylogenetic analyses have the following accession numbers: *Solanum lycopersicum* SlHQT (NP_001234850.1); *Solanum tuberosum* StHQT (NP_001275483.1); *Nicotiana tabacum* NtHQT (CAE46932.1); *Ipomoea batatas* IbHQT (BAA87043.1); *Coffea canephora* CcHQT (ABO77957.1); *Coffea arabica* CaHQT4 (AFP49814.1); *Coffea arabica* CaHQT (CAT00081.1)*; Platycodon grandiflorus* PgHQT (AEM63676.1); *Cynara cardunculus* HQT1 (ACF37072.1); *Cynara cardunculus* CcHQT2 (ADL62855.1); *Morus notabilis* MnHQT (XP_010094061.1); *Fagopyrum tataricum* FtHQT (AHA14500.1); *Salvia miltiorrhiza* SmHQT (ACA64049.1); *Ricinus communis* RcHQT (XP_002512739.1); *Medicago truncatula* MtHQT (KEH28560.1)*; Populus tomentosa* PtHQT (AFZ78609.1); *Populus trichocarpa* PtHQT (ACC63882.1); *Theobroma cacao* TcHQT1 (XP_007023475); *Arabidopsis thaliana* AtHQT (NP_199704.1).

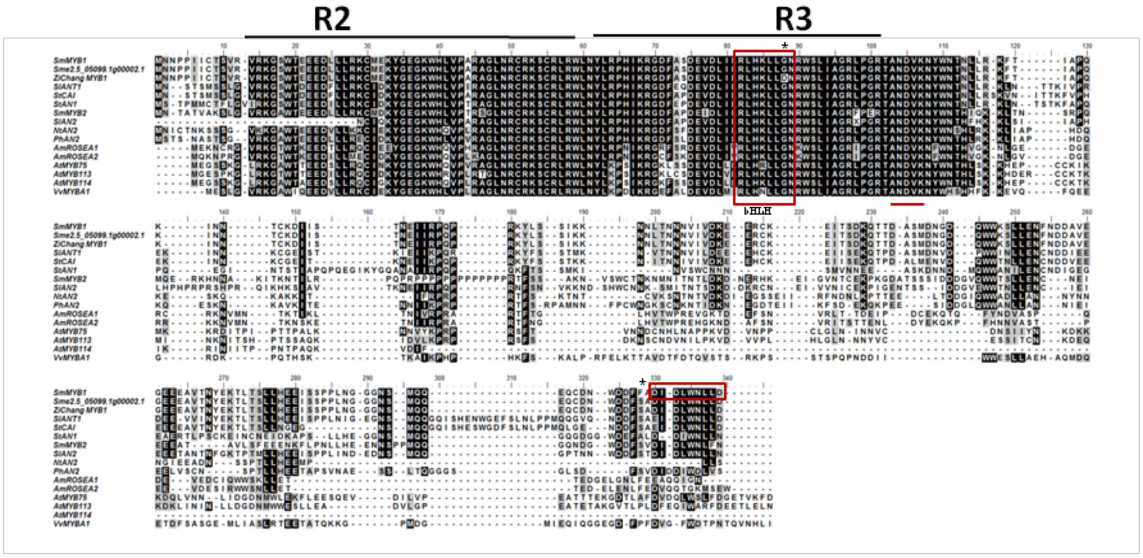

Supplementary Figure 3 The amino acid sequence of *S. melongena* Myb1 was aligned with the two MYB proteins from *S. melongena* Asiatic cultivars (SmMyb1 cv. 'ZiChang'; Sme2.5_05099.1_g00002.1 from the cv. 'Nakate-Shinkuro' Draft genome) and with 12 Myb representatives of other plant species including Solanaceae. The characteristic R2R3 domains are highlighted with black lines. Within them, a bHLH interacting domain and an ANDV motif are indicated in red by an open box and a solid line, respectively. A red open box at the C-terminal of the protein delimitates the 9 amino acids deletion in the *SmMYBΔ9* construct. Asterisks indicate aminoacid changes in respect to MYB1 from the cv. 'ZiChang' protein.

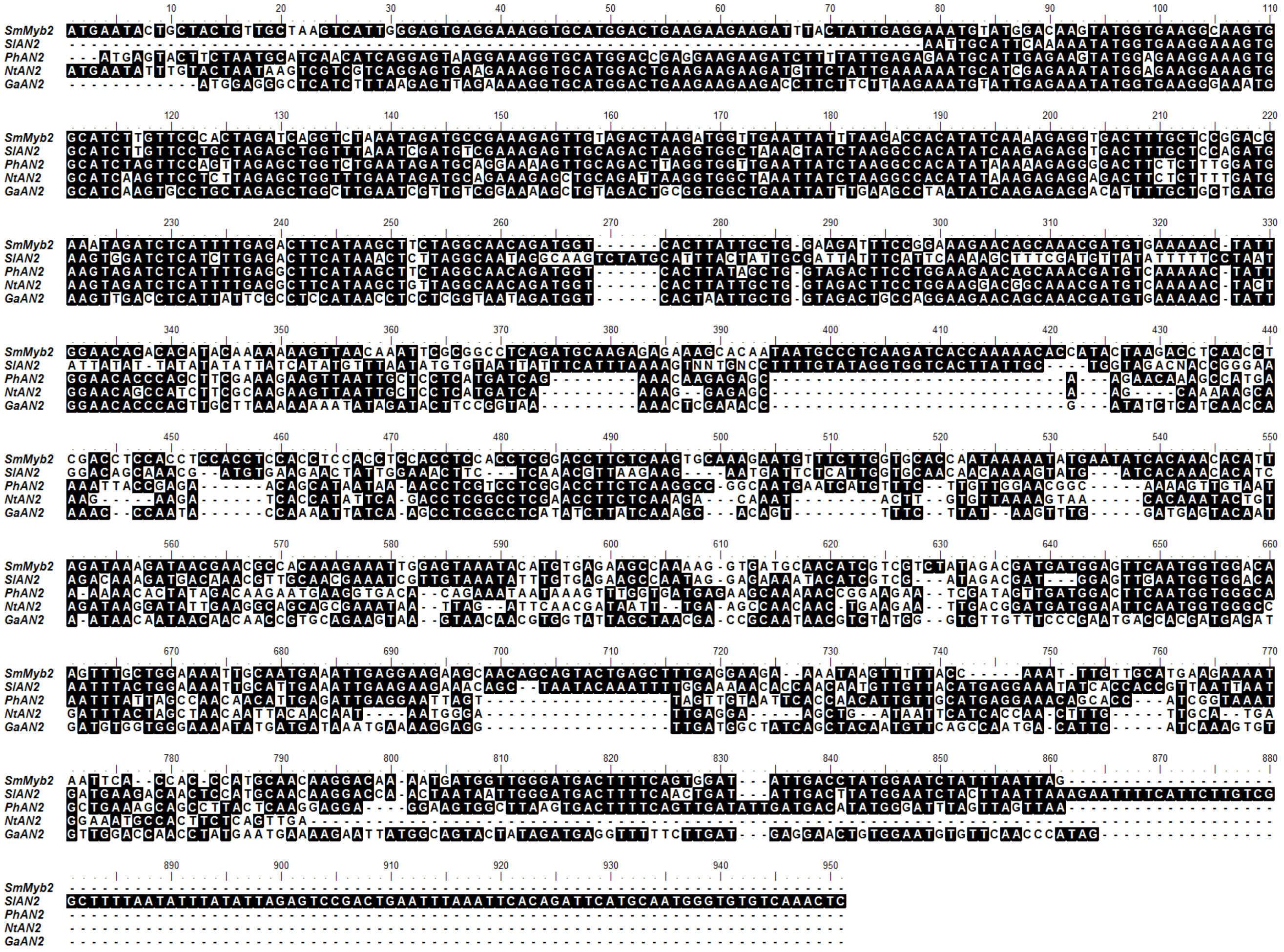

Supplementary Figure 4 The nucleotide sequence of *SmMyb2* (KF727477.1) was aligned with other highly similar AN2 genes from Solanaceae: *Solanum lycopersicum SlAN2* (FJ705333.1)*; Petunia hybrida PhAN2* (AB982128.1)*; Nicotiana tabacum NtAN2* (FJ472647.1); and with the AN2 gene from *Gossypium arboretum GaAN2* (KC491877.1).

(A)

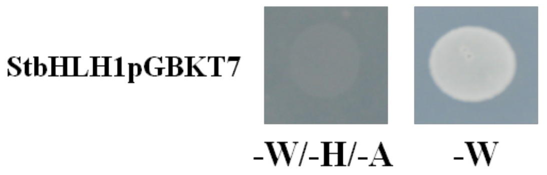

(B)

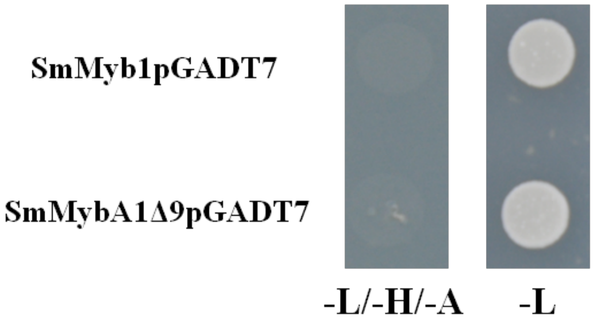

Supplementary Figure 5 Self activation-test. **(A)** *StbHLH1* cloned in the bait plasmid pGBKT7 (D'Amelia et al., 2014) was transformed in yeast and spotted on medium lacking tryptophan and on medium lacking adenine, histidine, tryptophan. **(B)** An equal amount of cells transformed with the prey plasmid pGADT7 containing *SmMyb1* or *SmMyb1Δ9* was spotted on medium lacking leucine and medium lacking adenine, histidine, leucine. The bait and prey plasmids when transformed alone conferred ability to grow on tryptophan or leucine, respectively, indicating presence of the plasmid, but not on media lacking three amino acids, which would have indicated self activation.
